# Molecular Profiling Defines Three Subtypes of Synovial Sarcoma

**DOI:** 10.1002/advs.202404510

**Published:** 2024-09-10

**Authors:** Yi Chen, Yanhong Su, Xiaofang Cao, Ioannis Siavelis, Isabelle Rose Leo, Jianming Zeng, Panagiotis Tsagkozis, Asle C. Hesla, Andri Papakonstantinou, Xiao Liu, Wen‐Kuan Huang, Binbin Zhao, Cecilia Haglund, Monika Ehnman, Henrik Johansson, Yingbo Lin, Janne Lehtiö, Yifan Zhang, Olle Larsson, Xuexin Li, Felix Haglund de Flon

**Affiliations:** ^1^ Division of Hematology and Oncology Department of Medicine Columbia Stem Cell Initiative Columbia University Irving Medical Center New York 10032 USA; ^2^ Department of Genetics and Development Vagelos College of Physicians and Surgeons Columbia University Irving Medical Center New York 10032 USA; ^3^ Program for Mathematical Genomics Department of Systems Biology Columbia University New York 10032 USA; ^4^ Department of Oncology‐Pathology Karolinska Institutet Stockholm 17177 Sweden; ^5^ Science for Life Laboratory Stockholm 17165 Sweden; ^6^ Faculty of Health Sciences University of Macau Taipa Macau 999078 China; ^7^ Department of Molecular Medicine and Surgery Karolinska Institutet Stockholm 17176 Sweden; ^8^ Department of Clinical Orthopedics Karolinska University Hospital Stockholm 17176 Sweden; ^9^ Department of Breast Cancer Endocrine Tumors and Sarcomas Karolinska University Hospital Stockholm 17176 Sweden; ^10^ College of Veterinary Medicine Northwest A&F University Yangling Shaanxi 712100 China; ^11^ Division of Hematology‐Oncology Department of Internal Medicine Chang Gung Memorial Hospital at Linkou Chang Gung University College of Medicine Taoyuan 33305 Taiwan; ^12^ Department of Clinical Pathology and Cancer Diagnostics Karolinska University Hospital Solna 17176 Sweden; ^13^ Department of General Surgery The Fourth Affiliated Hospital China Medical University Shenyang 110032 China; ^14^ Key Laboratory of Precision Diagnosis and Treatment of Gastrointestinal Tumors Ministry of Education China Medical University Shenyang Liaoning 110122 China; ^15^ Institute of Health Sciences China Medical University Shenyang Liaoning 110122 China; ^16^ Department of Physiology and Pharmacology Karolinska Institute Solna Stockholm 17165 Sweden

**Keywords:** BAF complex, copy number alterations, multi‐omics, synovial sarcoma, transcriptomics

## Abstract

Synovial Sarcomas (SS) are characterized by the presence of the SS18::SSX fusion gene, which protein product induce chromatin changes through remodeling of the BAF complex. To elucidate the genomic events that drive phenotypic diversity in SS, we performed RNA and targeted DNA sequencing on 91 tumors from 55 patients. Our results were verified by proteomic analysis, public gene expression cohorts and single‐cell RNA sequencing. Transcriptome profiling identified three distinct SS subtypes resembling the known histological subtypes: SS subtype I and was characterized by hyperproliferation, evasion of immune detection and a poor prognosis. SS subtype II and was dominated by a vascular‐stromal component and had a significantly better outcome. SS Subtype III was characterized by biphasic differentiation, increased genomic complexity and immune suppression mediated by checkpoint inhibition, and poor prognosis despite good responses to neoadjuvant therapy. Chromosomal abnormalities were an independent significant risk factor for metastasis. KRT8 was identified as a key component for epithelial differentiation in biphasic tumors, potentially controlled by OVOL1 regulation. Our findings explain the histological grounds for SS classification and indicate that a significantly larger proportion of patients have high risk tumors (corresponding to SS subtype I) than previously believed.

## Graphical abstract

By using transcriptomics and copy number profiling, synovial sarcomas are classified into three molecularly distinct classes defined by differences in hyperproliferation, genome instability and tumor immunology. These groups differed in response to chemotherapy and patient outcome, offering novel insights into the biology underlying the aggressive behavior of synovial sarcoma.

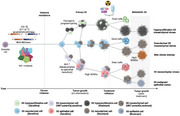

## 1 Introduction

Synovial sarcoma (SS) is an aggressive mesenchymal soft tissue malignancy. It represents the fourth most common type of soft tissue sarcoma, accounting for 5%–10% of all primary soft tissue sarcoma.^[^ ^1^, ^2^, ^3^, ^4^ ^]^ All SSs are characterized by a chromosomal translocation t(X;18) (p11; q11). This rearrangement results in a fusion between the SS18 gene on chromosome 18 (encoding a member of the chromatin modeling BAF complex) and one of three homologous genes *SSX1*, *SSX2*, or *SSX4* on chromosome X that form the fusion protein *SS18::SSX*.^[^ ^5^, ^6^, ^7^, ^8^ ^]^ The chimeric oncoprotein *SS18::SSX* hijacks the BAF complex and consequently reprograms chromatin architecture to promote sarcomagenesis,^[^ ^9^, ^10^, ^11^, ^12^, ^13^, ^14^ ^]^ essentially gaining stem‐cell‐like properties. Histologically SS can appear as monophasic (purely mesenchymal) or biphasic (mesenchymal and epithelial). These two subtypes are almost equally distributed (55% versus 45%, respectively).^[^ ^15^, ^16^ ^]^ Cases with increased nuclear atypia, necrosis, and higher mitotic index can be classified as poorly differentiated variants.^[^ ^15^, ^16^ ^]^

SS is relatively chemosensitive compared to other soft tissue sarcomas, but the efficacy of conventional therapeutic agents seems limited in patients with metastatic disease.^[^ ^17^, ^18^, ^19^, ^20^, ^21^ ^]^ Immunologically, SS is considered to be a poorly immunogenic type of cancer, and immune checkpoint inhibitors (ICIs) have thus far shown a low response rate in the treatment of SS.^[^ ^22^, ^23^, ^24^, ^25^ ^]^ SS homogeneously expresses multiple immunogenic cancer‐testis antigens (CTAs),^[^ ^26^, ^27^, ^28^ ^]^ however, the level of T‐cell infiltration within these tumors is insufficient for a robust immune response against these antigens.^[^ ^19^ ^]^ Single cell sequencing of SS identified a transcriptome signature coined the SS *core oncogenic program* ^[^ ^19^ ^]^ which was associated with poor prognosis, anti‐immunity, and aggressive disease. Genes in this signature were found to be frequently amplified in multiple SS cohorts.^[^ ^29^ ^]^ However, our understanding of the genomic events underlying phenotypic diversity in SS remains incomplete.^[^ ^30^, ^31^, ^32^ ^]^

Here, we apply multi‐omic analysis on a large cohorts of patients diagnosed at our institution over the last 30 years to provide a comprehensive molecular characterization of SS tumors.

## 2 Results

### 2.1 Identification of Three Molecular Subtypes of SS

**Figure 1:**
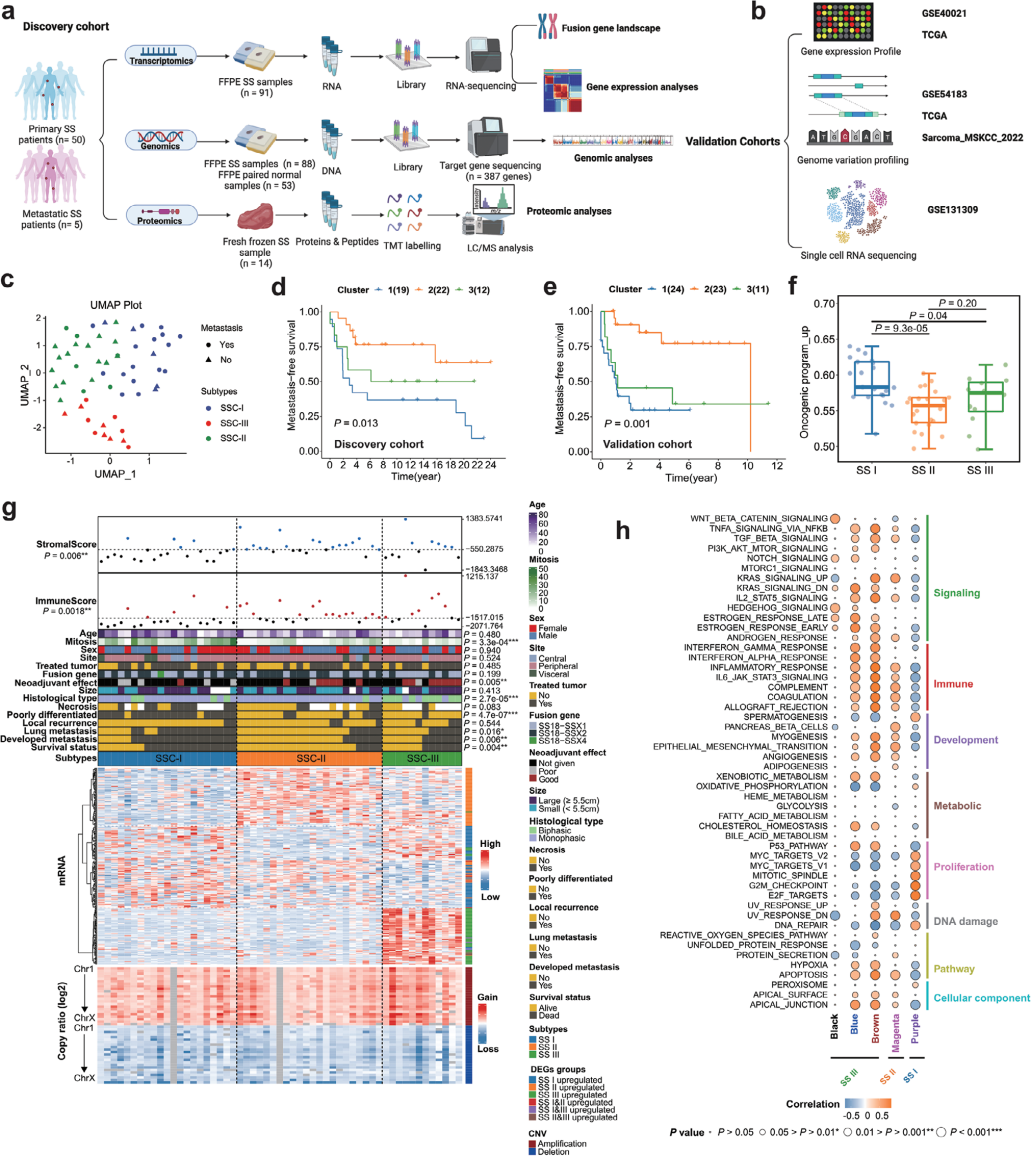
Synovial sarcoma subtypes and functional annotations a,b) The study workflow, including a) discovery and b) validation cohorts. c) UMAP plot of SS subtypes and metastatic status distributions. d) Kaplan‐Meier curve of clusters of metastasis‐free survival in discovery cohort at top 14% mRNAs variance (Log‐rank test, *P* = 0.013). e) Kaplan‐Meier curve of clusters of metastasis‐free survival in patients at top 14% gene variance applied at GSE40021 validation cohort (Log‐rank test, P = 0.01). f) Distributions of the ssGSEA score of upregulated genes in the oncogenic program across three SS subtypes. Middle line: median; box edges: 25th and 75th percentiles. Mann‐Whitney U test. g) Heatmap of the three SS subtypes. Clinicopathological characteristics (top) of the 55 synovial sarcoma patients are shown in the annotation, and different colors represent the characteristics and subtypes. Molecular data (bottom) from 55 patients profiled with mRNA and copy number variations are depicted. The statistical differences in categorical variables with three subtypes were compared using Fisher's exact test; Continuous variables were compared using the Kruskal‐Wallis test. *p < 0.05, **p < 0.01, ***p < 0.001. h) Association of five gene modules with 50 Hallmarks on ssGSEA scores. Spearman rank correlation. The shade of the color represents the levels of correlations, the size of circles represents the *p‐*value.

To unveil the tumor heterogeneity of synovial sarcoma (SS), we performed non‐negative matrix factorization (NMF) approach based on the transcriptomic data of mRNAs in 50 primary and five metastatic patients (Figures S2 and S3, Supporting Information). Transcriptome analysis revealed three major clusters of SS (named SS subtype I, II and III), that were also separated in the Uniform Manifold Approximation and Projection (UMAP) space (**Figure** **1**a–c).

SS subtype I was significantly associated with poor survival (Fisher's exact test, *P* = 0.004), metastasis (Fisher's exact test, *P* = 0.006), and the highest frequency of mitotic activity (*P* = 3.3e‐04). SS subtype II emerges as the archetype of a favorable prognosis, characterized by a scarcity of metastasis and mortality events during the follow‐up period. This subtype predominantly encompasses monophasic cases (Fisher's exact test, *P* = 2.7e‐05). On the contrary, SS subtype III primarily comprised biphasic cases, with a significant portion of patients demonstrating a profound response to neoadjuvant treatments (Fisher's exact test, *P* = 0.005), However, the majority of these patients experienced a relapse, leading to unfavorable outcomes. (Figure 1g) There was no statistically significant relationship (*P* > 0.05) between SS subtypes and age, sex, site, fusion gene type, tumor size, or local recurrence (Fisher's exact test, *P* > 0.05).

We then examined the top 14% most variable genes in the discovery and validation cohorts (Lagarde P et al. dataset^[^ ^31^ ^]^), which had consistent characteristics where cluster 1 (blue) denotes the worst prognosis, and cluster 2 (orange) represents the low‐risk group (log‐rank test, *P* = 0.013 and 0.001, respectively) (Figure 1d,e; Figure S6a and b, Supporting Information). The genes in the *SS core oncogenic program* (Table S6, Supporting Information) were significantly higher in SS subtype I, followed by subtype III and II (Figure 1f). These findings were consistently validated in Lagarde P et al. dataset (Figure S6c and d, Supporting Information). As expected, SS subtype I had the highest and lowest scores of proliferative and immune hallmark gene sets, respectively. Interestingly, SS subtype III had a significantly higher expression of IFN‐alpha and IFN‐gamma signatures (Figure S6e and f, Supporting Information). Functional enrichment of the 50 most differentially expressed genes between the subtypes (logFC >1.5, adjust *P*‐value < 0.01) highlighted expected biological processes, including proliferation, extracellular matrix composition, cytokine signaling and epithelial differentiation. (Figure S7a–c, Supporting Information). Similarly, gene set enrichment analysis (GSEA) identified significant enrichment of proliferation (e.g., E2F transcription factor activity, cell cycle and mitosis, normalized *P* < 0.001, FDR < 0.1) in metastatic patients (independently of SS subtypes), further strengthening the association between proliferation and metastasis. (Figure S8a–d; Table S7, Supporting Information).

To delve deeper into the intricacies of the subtypes, we engaged in a meticulous exploration of gene co‐expression networks, identifying distinct modules through the algorithmic framework of Weighted Gene Co‐expression Network Analysis (WGCNA) (Figure S9a–f, Supporting Information). The functional enrichment analysis of the genes within each module revealed striking concordances with the DEGs analysis. Functional analysis and ssGSEA (Single revealed upregulation of cell cycle and DNA damage response in SS subtype I, vasculature and endothelium development in SS subtype II, and epithelial cell differentiation, Wnt‐signaling and IFN‐alpha and IFN‐gamma signaling in SS subtype III (Figure 1g,h; Figure S10, Supporting Information).

### 2.2 Proteomic Validation of Gene Expression Findings

**Figure 2:**
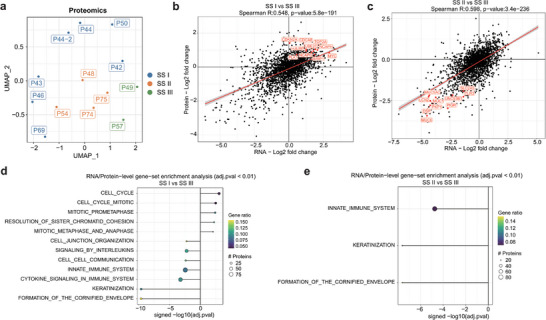
Proteomics data of three SS subtypes a) UMAP plot of SS subtypes in the proteome level. b,c) The correlation of DEGs between RNA and protein levels, Spearman rank correlation. d,e) The functional enrichment between d) SS I, e) SS II, and SS III in the RNA/protein level. Gene ratio: In the enrichment analysis, the number of gene symbols (overlapping RNAs and proteins) at the leading edge is divided by the total number of gene symbols in the given pathway. The size of the circles represents the number of enriched proteins at the leading edge.

Validating gene expression findings at the proteomic level is crucial for confirming the functional relevance of transcriptomic data and ensuring that observed changes in mRNA levels are reflected in protein expression. At the proteomic level, among the 13 viable samples, 2 were classified as SS subtype III, 7 as SS subtype I, and 4 as SS subtype II. These subtypes were distinctly delineated within the UMAP space, as depicted in **Figure** **2**a. A robust and significant correlation was observed between the levels of Differentially Expressed Genes (DEGs) and Differentially Expressed Proteins (DEPs) (Figure 2b,c). SS subtype I showed a significant overrepresentation of proteins linked to cell cycle pathways (Figure 2d), while SS subtype III was markedly abundant in proteins pertaining to innate immunity pathways (Figure 2e). Hence, the proteomic data corroborates the patterns observed at the gene expression level.

### 2.3 Mutational and Copy Number Landscape of SS Subtypes

#### 2.3.1 Genetic Alterations

**Figure 3:**
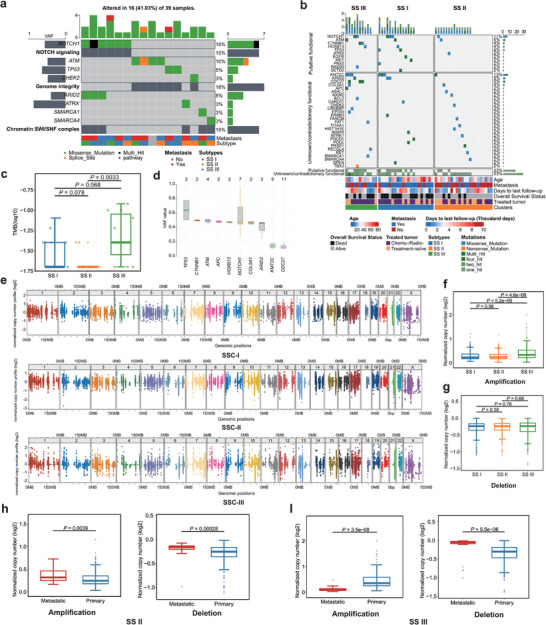
Genomic landscape of three SS subtypes a) Top three enrichment pathways of mutated genes and annotated below with metastasis and subtypes b) Distributions of the tumor mutation burdens (per 50 MB, logarithmic scale) in the three SS subtypes. Middle line: median; box edges: 25th and 75th percentiles. Mann‐Whitney U test. c) Distributions of variant allele frequency in mutated genes. Middle line: median; box edges: 25th and 75th percentiles. d) Distributions of variant allele frequency in mutated genes. Middle line: median; box edges: 25th and 75th percentiles. e) Chromosome scatter plots depicting the normalized copy numbers of three SS subtypes. f,g) The normalized copy number distributions of f) amplification and g) deletions in the three SS subtypes. Middle line: median; box edges: 25th and 75th percentiles. Mann‐Whitney U test. h) Normalized copy number distributions of amplifications and deletions between primary and metastatic in SS II patients. Middle line: median; box edges: 25th and 75th percentiles. Mann‐Whitney U test. i) Normalized copy number distributions of amplifications and deletions between primary and metastatic in SS III patients. Middle line: median; box edges: 25th and 75th percentiles. Mann‐Whitney U test.

Genetic alterations linked to oncogenesis (including enrichment of the NOTCH pathway, Genome Integrity and Chromatin SWI/SNF complex) were mostly frequently identified in SS subtypes III and I, respectively (**Figure** **3**a,b). However, the mutations in individual genes could not clearly distinguish between the subtypes (Figure 3a). SS subtype III was characterized by a significantly greater tumor mutational burden (TMB) than subtype II (*P* = 0.0033) (Figure 3c), and the highest level of DNA copy number alterations among the three subtypes. Importantly, our analysis revealed no discernible differences in mutational patterns between primary tumors and metastatic sites, nor did it demonstrate any correlation between mutational status and the overall survival of patients (Figure S11a and b, Supporting Information).

#### 2.3.2 Somatic Mutations

Consistent with previous studies,^[^ ^33^, ^34^ ^]^ the tumor mutational burden (TMB) within SS was found to be notably low (Figure S11c, Supporting Information). Overall somatic mutations were detected in tumors from 39 out of 53 patients, with a median of one SNV per sample. The majority of these SNVs were classified as missense mutations (Figure S11d, Supporting Information). Mutations in KMT2C were identified in 21% of patients, but the low variant‐allele frequency suggests that it may not serve a significant role as a clonal driver (Figure 3d). Other genes that were frequently mutated included NOTCH1 (15%), ATM (10%), and TP53, CTNNB1, and HOXB13, each with a frequency of 5% or less (Figure 3d; Figure S11d, Supporting Information). We cross‐referenced our mutation profile against previous studies using the TCGA (The Cancer Genome Altas Program) and Sarcoma_MSKCC_2022 cohorts, identifying four mutations that have been reported before: AXIN2 p.D159Y, COL5A1 p.R1133Q, CTNNB1 p.S45P, and PAX5 p.R117Q (Table S8, Supporting Information).^[^ ^30^, ^35^ ^]^

#### 2.3.3 Somatic Copy Number Alterations (SCNAs)

**Figure 4:**
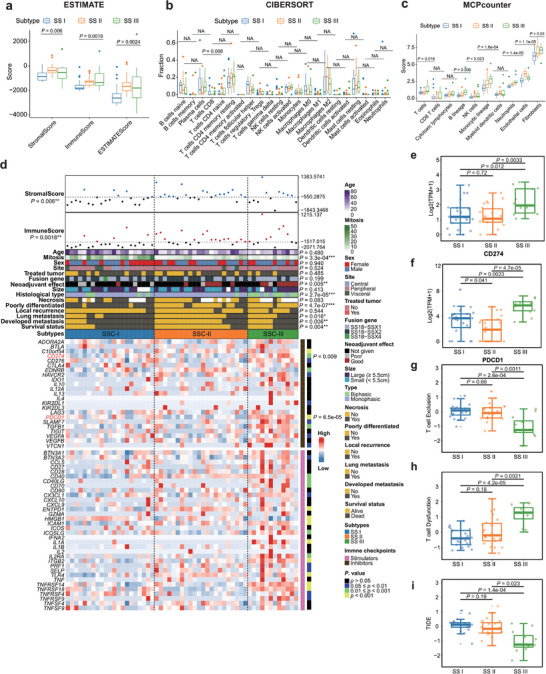
Tumor microenvironment of SS subtypes: a–c) The distributions of immune and stroma cell types across SS subtypes by three deconvolutional approaches, including a) ESTIMATE, b) CIBERSORT, and c) MCPCounter, respectively, Middle line: median; box edges: 25th and 75th percentiles. Kruskal‐Wallis test. d) Heatmap of the three SS subtypes on the mRNA levels of 23 inhibitory and 36 stimulatory immune checkpoints. Clinicopathological characteristics (top) of the 55 SS patients are shown in the annotation, and different colors represent the characteristics and subtypes. The statistical differences in categorical variables were compared using the Fisher's exact test; Continues variables were compared using Kruskal‐Wallis test. *p < 0.05, **p < 0.01, ***p < 0.001. e,f) Distributions of the gene expressions of *CD274* e) and *PDCD1* f) in three synovial sarcoma subtypes. Middle line: median; box edges: 25th and 75th percentiles. Mann‐Whitney U test. g–i) Distributions of the T cell exclusion, dysfunction, and TIDE score in three SS subtypes. Middle line: median; box edges: 25th and 75th percentiles. Mann‐Whitney U test.

We observed a significantly higher frequency of amplification in the genome‐wide SCNA profiles of SS subtype III compared to subtypes I and II (Figure 3e–g). In paired primary‐metastasis analysis of SS subtype II, metastatic lesions displayed significantly higher levels of amplification (*P* = 0.0039) and deletion (*P* = 0.00028) (Figure 3h), however this relationship was opposite in SS subtype III (*P* = 3.5e‐08) (**Figure** **4**i), indicating different mechanisms of clonal selection in metastasis progression. However, in SSC‐I, the difference was less significant (*P* = 0.53 in amplification; *P* = 0.48 in deletion) (Figure S12a, Supporting Information). In MP tumors, primary samples had significantly more deletions, while BP tumors showed a non‐significant increase in SCNA amplifications compared to SSC‐III (Figure S12b and c, Supporting Information). Analysis of a publicly available cohort (GSE54183) confirmed that chromosomal arm‐level copy number changes were significantly associated with risk of later metastasis (*P* = 4.5e‐11 for amplification; *P* = 0.015 for deletion) or metastasis at diagnosis (*P* = 0.00012 for amplification) (Figure S12d–g, Supporting Information).

### 2.4 SS Subtypes have Distinct Tumor Microenvironments

The tumor microenvironment (TME) of SS was investigating using RNA‐expression signatures associated with specific immune cells (ESTIMATE). Compared to other cancer types, SS showed exceptionally low immunological components (Figure S13a, Supporting Information). A comparative assessment between metastatic and primary SS tumors revealed minimal significant variations in immune and stromal cell profiles (Figure S13b–d, Supporting Information). Upon evaluating the immune profiles within the three SS subtypes, we found SS subtype III exhibited the most substantial immune cell infiltration, particularly neutrophilic granulocytes. SS subtype II was characterized by a significantly higher endothelial cell signature (Figure 4a–c). Across all subtypes, the infiltration of CD8+ cytotoxic T cells was consistently minimal.

We scrutinized the expression patterns of 59 immune checkpoints within the Synovial Sarcoma subtypes, distinguishing between 23 inhibitory and 36 stimulatory genes. Notably, SS subtype III had overexpression of inhibitory immune checkpoints which can be targeted with checkpoint inhibitors (i.e., *PDCD1* (*PD‐1*) and *CD274* (*PD‐L1*)) (*P* = 6.5e‐05 and *P* = 0.009, respectively) as displayed in Figure 4d–f. Subsequent analysis with the TIDE algorithm to evaluate the potential clinical effects of immunotherapy revealed high T cell dysfunction score in SS subtype III compared to subtype I and II (Figure 4g–i). The results showed that SS subtype III had a significantly elevated T cell dysfunction score when compared with subtypes I and II, also suggest a distinct immune evasion strategy within SS subtype III and imply that patients with this subtype might exhibit greater responsiveness to therapeutic agents aimed at PD‐1 or PD‐L1 inhibition.

### 2.5 SS Epithelial Components are more Susceptible to Chemotherapy Treatment

**Figure 5:**
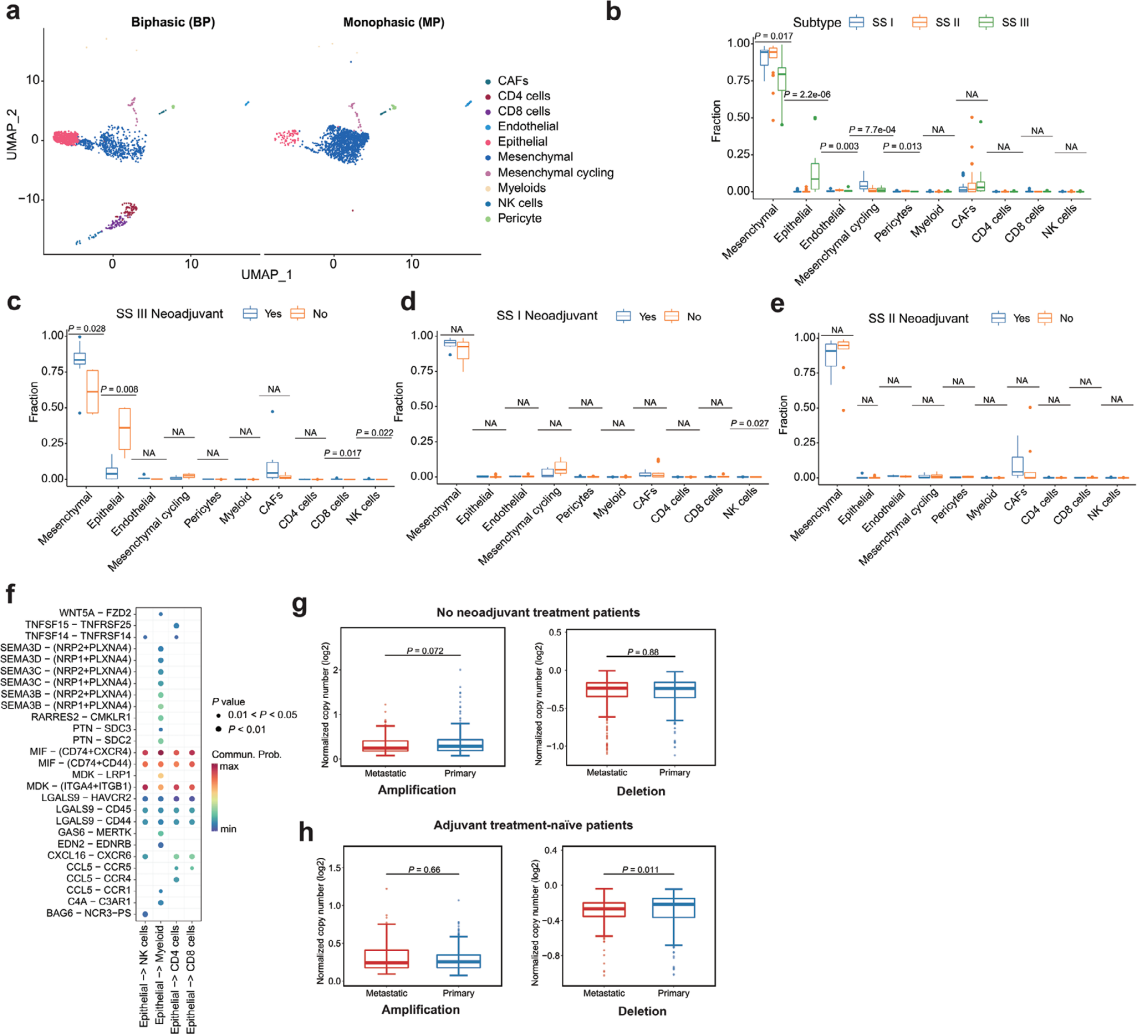
Treatment effects of SS subtypes a) UMAP plot of scRNA‐seq profiles in BP and MP tumors, colored by cell types. b) The distributions of cell types in three SS subtypes after CIBERSORTx deconvolution. Middle line: median; box edges: 25th and 75th percentiles. Kruskal‐Wallis test. c–e) Cell type distributions in neoadjuvant and no neoadjuvant treated SS III c), SS I d), and SS II e) patients after CIBERSORTx deconvolution. Middle line: median; box edges: 25th and 75th percentiles. Mann‐Whitney U test. f) Ligand–receptor pairs between epithelial cells and immune cells (NK cells, myeloid cells, CD4 cells, and CD8 cells. g) Normalized copy number distributions of amplifications and deletions between primary and metastatic in no neoadjuvant treatment patients. Middle line: median; box edges: 25th and 75th percentiles. Mann‐Whitney U test. h) Normalized copy number distributions of amplifications and deletions between primary and metastatic in adjuvant treatment‐naïve patients. Middle line: median; box edges: 25th and 75th percentiles. Mann‐Whitney U test.

To validate our results at single cell resolution we used publicly available dataset.^[^ ^19^ ^]^ For consistency we selected biphasic and monophasic tumors for downstream analysis (see Method, **Figure** **5**a; Figures S5 and S14a, Supporting Information). Utilizing this single‐cell dataset, we created a SS‐specific gene signature^[^ ^36^ ^]^ which was determined in our bulk RNA‐seq. As expected, SS subtype II was predominantly composed of endothelial cells, SS subtype III was enriched with epithelial cells, and SS subtype I was characterized by a significant presence of mesenchymal cycling cells (Figure 5b). In SS subtype III, neoadjuvant treatment led to a lower proportion of epithelial cells as compared to untreated tumors, indicating that cells with epithelial differentiation are more susceptible to chemotherapy treatment (Figure 5c). Complementing these observations, monophasic tumors did not exhibit any substantial shifts in cell composition across SS subtypes I and II, highlighting a consistent cellular makeup in these tumor subtypes (Figure 5d,e). Next, we examined the ligand‐receptor interactions between malignant cells and infiltrating T‐ and myeloid cells. Epithelial and mesenchymal cycling cells highly expressed the ligand Macrophage Migration Inhibitory Factor (MIF) and its receptors CD74, CXCR4, and CD44 in the immune cells (Figure 5f; Figure S14b and c, Supporting Information). Genomically, we observed where untreated tumors had fewer mutations than those receiving treatment, hinting at therapy‐induced mutagenesis (Figure S14d and e, Supporting Information). In patients without neoadjuvant therapy, primary tumors showed marginally more SCNAs than metastatic ones, suggesting clonal selection through adjuvant treatment and metastasis (Figure 5g). In untreated low‐risk patients, metastatic lesions had higher SCNAs, suggesting at the role of SCNA in tumor subclone evolution without chemotherapy (Figure 5h).

### 2.6 OVOL1 and KRT8 may Determine Epithelial Transition of SS Cells

**Figure 6:**
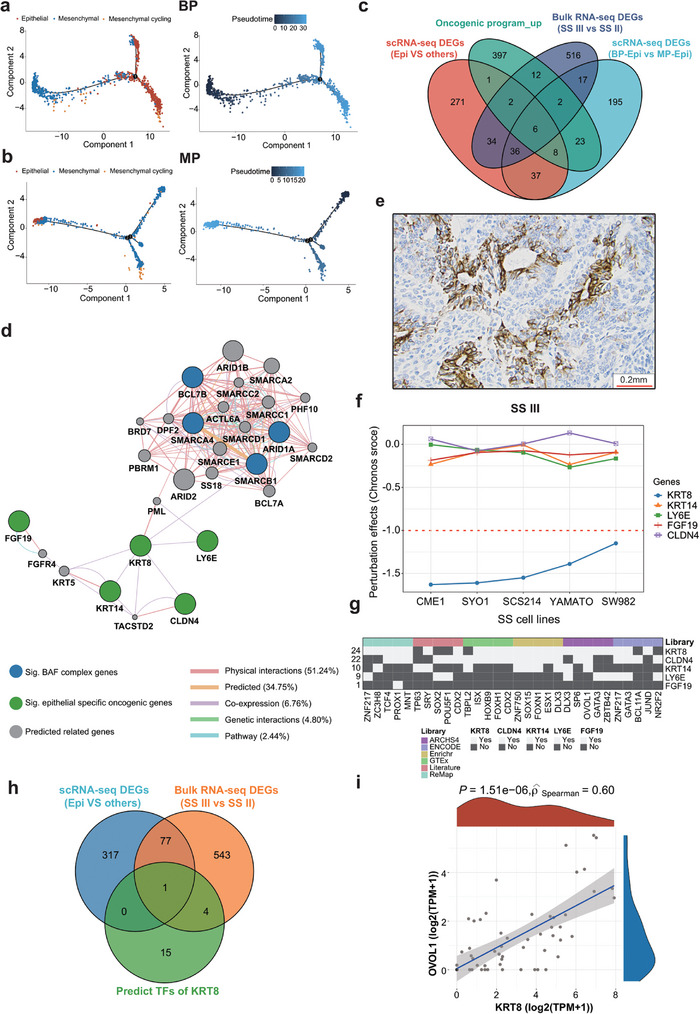
The regulation of mesenchymal to epithelial transitions in SS. a,b) The trajectory plots showing the dynamic development of epithelial, mesenchymal, and mesenchymal cycling cells in a) BP and b) MP patients and their pseudotime curve. c) Venn diagram depicting the overlap of the MET key genes involved in oncogenic program upregulated genes, Bulk RNA‐seq DEGs (SS III versus SS II), scRNA‐seq DEGs (BP‐epithelial versus MP‐epithelial), and scRNA‐seq DEGs (epithelial versus other cells). d) The predicted gene interaction network between significant BAF complex genes, epithelial specific oncogenic genes, and other putative additional relevant genes. e) KRT8 immunoreactivity in moderate scoring of SS III(T68). f) Perturbation effects of knockdown MET key genes in five SS cell lines. g) Schematic overview of predicted TFs of MET key genes. h) Venn diagram depicting the overlap of the MET key genes involved in predicted TFs of KRT8, Bulk RNA‐seq DEGs (SS III versus SS II), and scRNA‐seq DEGs (epithelial versus other cells) i) The correlation between expression levels of KRT8 and OVOL1, Spearman rank correlation.

Trajectory plots were generated from SS scRNA‐seq data^[^ ^19^ ^]^ and a revealed substantial transition from mesenchymal to epithelial cells in biphasic tumors, but less noticeable in MP patients (**Figure** **6**a,b). By comparing DEGs from bulk RNA‐seq (SS subtype III versus subtype II) and scRNA‐seq (biphasic versus other cell types), as illustrated in Figure 6c, six genes were found overexpressed in the epithelial cells of biphasic tumors (*KIF1A*, *KRT8*, *CLDN4*, *KRT14*, *LY6E*, and *FGF19)*. The expression of these genes all correlated well with mesenchymal to epithelial transition in individual trajectory plots (Figure S15a and b, Supporting Information). Our research then narrowed in on KRT8 due to its putative direct interaction with the BAF complex, setting it apart from the other pivotal genes (e.g., *LY6E, CLDN4, KRT14*, and *FGF19*, which seem to orchestrate the downstream regulatory pathways (Figure 6d). KRT8 protein expression was confirmed specifically in SS subtype III by immunohistochemistry (Figure 6e; Figure S16, Supporting Information). Data from the Dependency Map portal^[^ ^37^, ^38^ ^]^ showed that *KRT8* is essential for SS cell survival in vitro. (Figure 6f). By using the ChEA3 database^[^ ^39^ ^]^ we identified 24 potential transcription factors regulating *KRT8*, but only *OVOL1* had significant co‐variation with *KRT8* in bulk RNA and scRNA DEG (ρ = 0.60, *P* = 1.51e‐06) (Figure 6g–i). Taken together, *KRT8* is associated with epithelial differentiation in biphasic SS biology and could be regulated by the transcription factor *OVOL1*.

### 2.7 Fusion Gene Landscape in SS

Gene fusions were detected in all 91 RNA‐sequenced samples from 55 patients by the FusionCatcher (primary) and STAR‐Fusion (supportive) pipelines. Prior to filtering there were 9750 candidate fusion transcripts with the FusionCatcher tool across all the samples, with *SS18::SSX* detected in all but three samples (Figure S4, Supporting Information).

The common *SS18::SSX* fusions were present in most of the cases, whereas a novel *SS18::SSX2* fusion variant was detected involving the traditional breakpoint in *SS18* transcripts (…ATATGACCAG*) and novel *SSX2* (*GACCCAAAG…) on ENST0000033677. As expected, the *SS18::SSX1* and *SS18::SSX2* variants predominated, and the *SS18::SSX4* occurred in only seven samples (Figure S17a, Supporting Information). Next, we assessed the expression level of the fusion gene in ten patients with multiple samples in a longitudinal analysis. We found inconspicuous changes in *SS18, SSX1*, and *SSX2* expression after treatment or metastasis (Figure S17b, Supporting Information). Our data also supported that there was no significant difference between SSX1 and SSX2 fusions in terms of overall survival (OS) or metastasis‐free survival (MFS) (log‐rank test, *P* = 0.637 and 0.494, respectively) (Figure S17c, Supporting Information), similar to the patient outcomes in the validation cohort (log‐rank test, *P* = 0.498, Figure S17d, Supporting Information).

To explore the biological significance of secondary fusion genes, we performed a filtering process (see Methods, Figures S4 and S18a,b, Supporting Information) to obtain whitelisted fusion genes for further validation at the molecular level. No secondary fusion genes were associated with the SS18 or SSX chromosomal regions (Table S2, Supporting Information). We found that 41 fusion genes arose from intrachromosomal rearrangements, while 98 of 139 fusion genes were potentially generated by interchromosomal translocations (Figure S18c, Supporting Information). Notably, in‐frame fusions comprised most whitelisted fusion pairs. We also investigated if the number of secondary fusion genes was associated with treatment or metastatic disease, but no significant associations could be found (Figure S18d and e, Supporting Information). RT‐PCR successfully validated 12 fusion genes (Figure S18f, Supporting Information), six of which were successfully cloned and sequenced, including two BAZ2B‐DNAH11 variants (Figure S4g, Supporting Information). Interestingly, the BAZ2B‐DNAH11, EDA‐KDM5C, NOR1‐SPAG9, and ZFHX4‐LRRC69 fusion genes had protein functions associated with chromatin remodeling. However, none of the whitelisted fusion genes were recurrent in our cohort (Figure S5a–f, Supporting Information), suggesting that secondary fusion events are rare, perhaps stochastic, and with limited clinical importance.

## 3 Discussion

In this study we identified three transcriptomic clusters of SS. These subtypes were characterized by distinct differences in histology, gene expression patterns, tumor microenvironment, DNA copy number alterations and clinical outcome.

### 3.1 SS Subtype I (Hyperproliferative)

Patients with SS subtype I had the worst prognosis. These tumors were distinguished by high expression of E2F and MYC targets functionally related to proliferation, DNA repair pathways and the previously described *core oncogenic program*.^[^ ^40^ ^]^ The lack of secondary genomic alterations in the form of mutations or copy number alterations suggests that this phenotype is defined *de novo*, perhaps as a result by the condition of the BAF‐complex composition. This was also supported by the absence of switching between SS subtypes in patients with multiple sequenced lesions at different time points. The observations of clonally evolutionary distinct subgroups are in line with the observations done by Przybyl et al.^[^ ^41^ ^]^ Interestingly, many tumors in the hyperproliferative‐SS group were not histologically characterized as poorly differentiated SS, suggesting that the histological criteria for classifying poorly differentiated SS should be reviewed with this in mind. Importantly, the high expression of the core oncogenic program driven by E2F and MYC targets activate pathways that enhance DNA repair and mimic stem‐cell like properties, contributing to the overall chemotherapy resistance observed in this subtype.

### 3.2 SS Subtype II (Hypervascularized)

The SS subtype II was characterized by better patient outcome (the 10‐ and 20‐year overall survival was 75%). These tumors displayed higher expression of stromal signatures, higher expression of vascular‐related genes, downregulation of DNA‐repair pathways and the lowest expression of the *core oncogenic program*. While most SS subtype I cases had non‐existent mutational burden and SCNA alterations, patients that died of disease in this group had distinctly higher frequency of copy number amplifications (Figure 1e) indicating that second hits might be required for progression and metastasis in this subtype. The overall lower proliferation and gene expression patterns suggestive of less active oncogenic signals may be the main reason these patients have better outcome. However, we cannot ascertain if the better outcome stems from a truly less aggressive phenotype, heightened chemotherapy sensitivity (many patients were given adjuvant therapy), or a synergy of both factors.

### 3.3 SS Subtype III (Epithelial)

SS subtype III predominantly featured biphasic tumors and was marked by elevated levels of kinase‐signaling targets, increased infiltration by innate immune cells, and diminished activity in proliferation and DNA repair pathways. Since this subtype had the highest frequency of copy number alterations and somatic mutations, it is reasonable to infer that these genomic alterations serve as defining drivers. Through expression network analysis, we identified a low molecular weight cytokeratin (KRT8) as a crucial gene for mesenchymal to epithelial transition. KRT8 maintains cellular structural integrity and participates in signal transduction and cellular differentiation.^[^ ^42^ ^]^ Furthermore, we hypothesize that OVOL1, a transcription factor known to critically regulate the differentiation of epithelial cells^[^ ^43^, ^44^ ^]^ might be key to KRT8 regulation. However, additional research is needed to elucidate the regulatory mechanisms that convert SS cells to an epithelial phenotype. Interestingly, although many tumors of this subtype exhibited an extensive histopathological response to neoadjuvant chemotherapy (<10% viable tumor cells), both the long‐term MFS and OS were markedly poor. The transcriptome profiling was able to determine that the epithelial component was proportionally lower in the neoadjuvant treated tumors, suggesting that the mesenchymal component is more chemo resistant. Given that metastatic lesions of these tumors exhibited biphasic pattern one could assume that the mesenchymal cells retain the property to transition to epithelial cells.

### 3.4 Validation and Immunological Insights

In our validation of the three SS subtypes, we cross‐referenced our transcriptomic data with the established Lagarde et al. dataset,^[^ ^31^ ^]^ we solidified our subtyping with a confirmatory backdrop of genetic consistency. We took this a step further by mapping our proteomic signatures to the established gene expression profiles, uncovering a synergistic relationship that supports to the authenticity of our findings. Furthermore, the use of scRNA‐seq data allowed us to refine our SS‐specific gene signature, underpinning the cellular heterogeneity within subtypes. This single‐cell resolution brought to light nuances in cellular composition and behavior, that were not previously captured in bulk RNA analyses. The mutations we mapped were consistent with the known genomic alterations in SS, but we delved more deeply into the subtle differences in mutation frequency post‐treatment. This not only validated the presence of treatment‐induced genomic changes but also provided a more detailed understanding of clonal evolution in response to therapeutic interventions.

From an immunological vantage point, our findings pierce through SS's historical portrayal as non‐immunogenic, unveiling potential immunotherapeutic avenues. Petitprez et al. identified five distinct immune classes for sarcoma. Interestingly, half of the synovial sarcoma instances were categorized under the “immune desert” phenotype, while the rest were grouped into either “immune‐high” or “highly vascularized” categories,^[^ ^45^ ^]^ which show striking similarity with our SS subtype classification. When juxtaposed with other cancers, SS stands out due to its reduced adaptive immune response, coupled with a pronounced presence of dormant B cells in their naïve state and impaired CD8^+^ T cells. But given that SS metastasis may develop after a very long time (10+ years), such clinical studies may be very difficult to execute. To date, clinical trials involving CTLA‐4 and PD‐1 inhibitors have shown limited efficacy in treating synovial sarcoma, with response rates falling below 10%.^[^ ^22^, ^23^, ^46^ ^]^ However, with the swift advancements in immune checkpoint inhibitor therapies, these findings pave the way for a more in‐depth exploration of potential immunotherapy for SS.

### 3.5 Genomic Alterations and Therapy Resistance

In line with previous studies, we found low levels of secondary mutations, and varying levels of chromosome complexity in SS. Not surprisingly, therapy naïve tumors that developed metastasis had higher levels of copy number alterations compared to tumors that did not develop metastasis, indicating that it might be important for tumor progression. Interestingly we observed the opposite relationship in tumors that had been treated with chemotherapy, suggesting that primary tumors may contain multiple clones and the post‐treatment metastasis with a less complex genome can be the consequence of clonal selection during therapy. We found that a substantial proportion of cases harbored mutually exclusive mutations in genes associated with genome integrity (18%) or the BAF complex (15%), but we were unable to find any association to clinical outcome or molecular phenotype. We also identified and validated non‐recurrent structural variants resulting in secondary fusion genes. While most of the validated fusion genes were predicted to produce functional proteins, we were unable to provide evidence for their role as oncogenic drivers. Since we were unable to identify any clinical relevance for secondary fusion genes, we conclude that they most likely constitute stochastic events with limited biological relevance in SS. However, we cannot rule out that individual fusion genes resulting in, i.e., constitutionally signaling fusion proteins could have immense importance for individual tumors, such events are probably very rare and should be investigated on an individual bases. Increased number of stochastic secondary fusion genes could be a consequence of an unstable genome and could potentially act as biomarker for this in the absence of DNA copy number profiling. However, this was not within the scope of this study.

### 3.6 Origins and Limitations

The origins of synovial sarcoma have long been debated, with theories suggesting roots in multipotent mesenchymal stem cells, myogenic progenitor cells, or synovial cells. The distinct gene expression patterns in the SS subtypes we identified could hint at varied cells of origin. On a purely speculative basis, the aggressive SS subtype I might stem from primitive progenitor cells; SS subtype II, with a vascular‐stromal signature, might originate from vascular or connective tissue lineage and SS subtype III, displaying both epithelial and mesenchymal traits, might suggest roots in an epithelial progenitor cell. Identifying the cell of origin for each SS cluster in future studies could shed light on this intricate disease's pathogenesis and help explain the phenotypical differences.

Although the bioinformatics approaches for analyzing bulk RNA and DNA sequencing data have been well established, this study has some inherent limitations. Our sample size was insufficient for robust longitudinal analyses to observe differences in metastatic evolution among SS subtypes, and the true predictive effect of the gene signature‐based classification needs prospective validation. While single‐cell RNA‐seq data integration provided insights, further refinement is needed to fully explore intra‐tumor heterogeneity. Additionally, bioinformatics challenges such as batch effects, data integration, and complex multi‐omics data interpretation require advanced computational methods. Future research should include larger cohorts with longitudinal data, prospective validation of gene signatures, and advanced single‐cell and spatial technologies to dissect tumor heterogeneity. Developing sophisticated computational tools and performing functional validation of findings through experimental approaches will enhance the understanding of SS and improve personalized therapeutic strategies.

## 4 Conclusion

To conclude, our results revealed a substantial heterogeneity among SS and facilitated patient stratification into three subtypes. We described the biological and genetic variability between the subtypes, including key transcriptome patterns, secondary genomic events, including copy number alterations which could be associated with the long‐term outcome. All SS exhibited an immune inhibitory phenotype, albeit with some differences including gene expression of immune checkpoint markers. We believe that these results explain parts of the biology underlying SS and will help us understand the differences in clinical presentation which may prove important for future therapy development and patient stratification.

## 5 Experimental Section

### Patient Samples and Ethics

The digital patient records of the Pathology Clinic at the Karolinska University Hospital were searched for cases of synovial sarcoma. Formalin‐fixed paraffin‐embedded (FFPE) tissue was collected from all cases classified as SS in the archive and was reviewed. In retrospect and without the detailed mapping of treatment protocol or pathology sampling protocol, primary tumors that had been treated with neoadjuvant treatment were classified as good responders when ≤10% of the tumor was deemed viable or as poor responders when >10% of the tumor was deemed viable. To include a case in the study, we required DNA or RNA with sufficient quality for sequencing and proof of the SS18::SSX fusion gene. Clinical FISH or RT‐PCR was available for most cases, but to further validate the diagnosis, all cases were confirmed positive for the SS18::SSX chimeric‐protein specific antibody (Figure S1a, Supporting Information). In the end, a total of 55 patients with SS were included in the study. Fifty patients had localized tumors, and five patients had metastatic disease at the time of diagnosis. All patients were surgically treated at the Karolinska University Hospital between 1992–2016, and the follow‐up data collection was concluded in December 2019.

A schematic presentation of the study design is represented in Figure 1a,b. Among the 55 SS patients, 88 tumor samples had both whole transcriptomic data (WTS) and target gene DNA‐seq data, of which 14 fresh frozen samples had proteomics data. As shown in Table S1a and b and Figure S1b and c (Supporting Information), seven of 21 patients (33%) that received neoadjuvant therapy had >10% viable tumor tissue (poor responder). A total of 28 (50.9%) patients developed distant metastases (median: 5.1 years, range: 0 – 24 years), of which 24 (43.6%) patients died of the disease during the follow‐up. Patients with metastasis were given chemotherapy and radiotherapy (16.7%), chemotherapy alone (29.2%), or radiotherapy alone (37.5%). The 5‐year overall survival rate was 50.9%, and the 10‐year overall survival rate was 41.5%, confirming the aggressive nature of SS.

### Validation Cohorts

For the validation groups, the microarray‐based gene expression profiles for 58 SS cases from GSE40021 was accessed,^[^ ^31^ ^]^ the single‐cell RNA sequencing (scRNA‐seq) gene expression profiles for 12 SS cases from GSE131309,^[^ ^19^ ^]^ and the chromosomal arm‐level variation data for 54 SS cases from GSE54183.^[^ ^29^ ^]^ All data sets, including the relevant clinical information, were retrieved from the NCBI Gene Expression Omnibus (GEO) database. Additionally, for genomic analysis, Sarsoma_MSKCC_2022 cohort^[^ ^35^ ^]^ was also used for validation the somatic copy number alterations (SCNAs) and single nucleotide variants (SNVs). For the pan‐cancer analysis, the uniformly normalized pan‐cancer dataset was downloaded: TCGA Pan‐Cancer (PANCAN, N = 10 535, G = 60 499) from the UCSC (https://xenabrowser.net/) database, the gene expression profile of each tumor was extracted separately and mapped to the genome annotation file (hg19). In addition, we performed log2 (x+1) transformation of each expression value. In terms of the genomic data, the Simple Nucleotide Variation dataset of the level4 of all TCGA samples processed by MuTect2^[^ ^47^ ^]^ software from GDC (https://portal.gdc.cancer.gov/) was obtained, and calculated the tumor mutation burden (TMB) of each tumor using the “tmb” function of the R package “maftools” (v 2.6.05),^[^ ^48^ ^]^ It was also excluded cancer types with less than three samples, and finally obtained the expression data of 33 cancer types.

### Tissue DNA and RNA Extraction

After histological review, areas with high tumor purity (range 95% – 75%) were dissected from FFPE blocks using 1 mm punch biopsies. Tumor total RNA and DNA were extracted from 91 lesions, where 55 were from primary tumors and 36 from metastatic lesions. Normal DNA was extracted from adjacent normal tissue (n = 88). A summary of the sample processing was shown in Figure 1a. RNA and DNA were isolated from the FFPE tissue samples with a separate punch biopsy using the QIAamp RNeasy FFPE Extraction Kit (Qiagen, Cat # 73504) and QIAamp DNA FFPE Tissue Kit (Qiagen, Cat # 56404), respectively, according to the manufacturer's instructions. For RNA, the quality was assessed using RIN (RNA Integrity Number) values and DV200 values using a Tapestation (Agilent) along with the A260/A280 ratio measured using a Nanodrop. Samples with RIN values greater than 7, DV200 values greater than 70% and A260/A280 ratios within the range of 1.8–2.1 were considered of sufficient quality. High‐quality DNA was characterized by an A260/A280 ratio within the range of 1.8–2.0, indicating pure DNA free from significant protein contamination. Additionally, we performed gel electrophoresis to assess DNA integrity. High‐quality DNA was considered as that which showed a tight band at a high molecular weight with minimal to no smearing, indicative of low degradation. After extraction, the RNA and DNA samples underwent next‐generation sequencing for library preparation and sequencing.

### Whole Transcriptomic Analysis

Library generation, quality control, sequencing, and initial data processing were performed at the National Genomics Infrastructure, Science for Life Laboratories in Stockholm. RNA library preparation was done using the Illumina TruSeq Stranded Total RNA and the Illumina RiboZero. Clustering was done by “cBot,” and samples were sequenced on NovaSeq6000 (NovaSeq Control Software 1.6.0/RTA v3.4.4) with a 2 × 151 setup using ‘NovaSeqStandard’ workflow in “SP” mode flowcell. The Bcl to FastQ conversion was performed using bcl2fastq_v2.19.1.403 from the CASAVA software suite. The quality scale used was Sanger / phred33 / Illumina 1.8. The average sequence depth was 37.5 M reads (min 16.4 – max 82.3 M reads).

### RNA seq Data Processing

RNAseq data were processed with the nf‐core RNAseq pipeline v1.0.^[^ ^49^ ^]^ Default parameters were used unless mentioned otherwise. Sequencing data quality was assessed using the FastQC.^[^ ^50^ ^]^ TrimGalore was used to remove adapter contamination and trim low‐quality areas (https://www.bioinformatics.babraham.ac.uk/projects/trim_galore/), with the Cutadapt function for adapter trimming and the FastQC function upon completion.^[^ ^51^ ^]^ Sequences were aligned to the human reference genome (hg19/GRCh37) by applying the STAR software. Transcript counts were produced with featureCounts^[^ ^52^ ^]^ using the transcriptome annotation file generated by StringTie.^[^ ^53^ ^]^ We reported the gene expression data in both counts and transcripts per million (TPM) values. Raw counts were used for differential expression analyses (“DESeq2” package^[^ ^54^ ^]^ in R). For quantification purposes, Transcripts per million (TPM) were calculated using Kallisto (version 0.46.1).^[^ ^55^ ^]^ Transcripts with a TPM score above one were retained, resulting in a total of 18 934 gene IDs. All known exons in the annotated file were 100% covered. Log transformation of TPM values was calculated as log2 (TPM + 1) and applied for subsequent analyses.

### Gene Network Construction and Functional Enrichment Analysis

Both Gene Set Enrichment Analysis (GSEA v4.0)^[^ ^56^, ^57^ ^]^ and Metascape^[^ ^58^ ^]^ were used for pathway enrichment analysis. Specifically, samples of all gene expression data (normalized) were pooled according to the paired grouping of patients and were subjected to GSEA. Molecular Signatures Database^[^ ^59^ ^]^ (MSigDB v7.4) of Hallmark gene sets (H), Reactome gene sets (C2), and Gene Ontology (GO) biological process gene sets (C5) were employed for enrichment analysis. Nominal *P*‐value <0.01 and FDR < 0.01 were selected as the threshold as significance. Additionally, Metascape was used to identify gene enrichment terms in up‐or down‐regulated genes for GO and Reactome pathway analyses. To identify gene expression patterns associated with the metastatic disease, it was performed five comparisons for GSEA: i) Comparison 1: all metastatic samples compared to all primary samples; ii) Comparison 2: Over follow‐up time in 10 patients with multiple lesions, metastatic samples compared to all primary samples; iii) Comparison 3: patients with metastases versus without; iv) Comparison 4: treatment‐naïve patients with metastases versus without; v) Comparison 5: patients with metastatic events versus without in a validation cohort‐GSE40021). Single‐sample gene set enrichment analysis (ssGSEA) was employed to calculate the sample enrichment score according to the given gene sets.^[^ ^60^ ^]^ Analysis for gene networks and pathways was performed with the Cytoscape v3.8.2 software^[^ ^61^ ^]^ with the GeneMANIA^[^ ^62^ ^]^ plugin according to the instruction manual. It was analyzed gene networks to identify gene‐to‐gene interactions, the topology of such gene correlations, and putative additional genes that may be involved in significant BAF complex genes and epithelial cell‐specific oncogenes if shown to interact with the large number of genes in the query set.

### Immune Profiling Analyses

It was performed seven different computational algorithms to assess the immune cell content in the tumor microenvironment (TME), and the results were confirmed by assessing the consistency of the abundance/fraction of immune cells in the cross‐validation of deconvolution methods. Cell‐type identification by estimating relative subsets of RNA transcripts (CIBERSORT)^[^ ^63^ ^]^ to quantify the fractions of the 22 immune cell subtypes was performed. In terms of immune cell abundance, the Estimation of Stromal and Immune cells in Malignant Tumor tissues using the Expression data (ESTIMATE) tool to determine the score of immune and stromal cells was utilized.^[^ ^64^ ^]^ MCPCounter was implemented to calculate the abundance of tissue‐infiltrating immune and stromal cell populations in 10 cell types.^[^ ^65^ ^]^ TIDE software with default parameters to analyze the tumor T cell dysfunction and exclusion scoring was used.^[^ ^66^ ^]^ To generate a SS specific gene signature matrix in Bulk‐RNA seq using immune cell types from the scRNA‐seq data, the Create Signature Matrix module (https://cibersortx.stanford.edu/runcibersortx.php)^[^ ^67^ ^]^ with the log‐normalized expression matrix from the dataset restricted to immune populations of interest supplied as a reference matrix was ran. It was used a Min. Expression parameter of 1, No. Barcode Genes parameter of 300 to 500, and q‐value at 0.01 with all other parameters set to default. Accordingly, CIBERSORTx deconvolution was performed on the SS bulk RNA‐seq cohorts in relative mode with S‐mode batch correction and quantile normalization disabled to re‐impute the cell fractions.

### Non‐Negative Matrix Factorization (NMF) Clustering

To identify new optimal molecular subtypes of SS, NMF clustering using the R “NMF” package was performed^[^ ^68^ ^]^ with the parameters as follows: K = 2–10, method = “brunet”, run = 50. Log2 transformed TPM data was used as the input data for clustering. Rather than separating clusters based on distance computation, NMF detects context‐dependent patterns of gene expression in complex biological systems. To generate the most robust clustering, 25 different truncation points were set from the top 6% gene variance to the top 30% gene variance (Figure S2a, Supporting Information). The cophenetic correlation coefficient to determine the clusters that yield the most robust clusters was used. The cophenetic correlation coefficient was calculated from the consensus matrix of NMF clusters. From the plot of the resonance correlation coefficient versus k, the top point where the coefficient of conjugate correlation decreased the most was chosen. The silhouette width was used to evaluate the performance of clustering, which was defined as the ratio of the average distance of each sample from samples in the same cluster to the minimum distance of samples not in the same cluster.

According to the optimal K numbers in each group, patients were classified into given groups (Figure S2b and c, Supporting Information). The optimal numbers of clusters were picked from each set, and it was calculated and then adopted three strategies to evaluate the metastasis‐free survival (MFS) rate across clusters. The strategies included: i) using the original subtypes delineated by NMF; ii) combining groups of three or fewer patients with the nearest prognostically aligned group, termed as outliers incorporated; and iii) excluding outliers from the MFS evaluation. A comparative analysis of p‐value significance across these strategies retained three clustering candidates (top 14%, top 16%, and top 23% variance) for further evaluation (Figure S3a–c, Supporting Information). As a result, compared to the top 16% variance and top 23% variance, the consensus matrix showed that the top 14% had less overlap between clusters. By thoroughly examined the inherent characteristics of these subgroups and their overarching similarities, as well as considerations of the biological and clinical relevance of the identified subgroups, the outliers was incorporated, no significant difference was seen in overall survival for both the top 16% (Figure S4d, Supporting Information) and the top 23% (Figure S4e, Supporting Information) variance groups (log‐rank test, *P* = 0.058 and 0.082, respectively), regardless of the significance of MFSs (log‐rank test, *P* < 0.05) (Figure S4g and h, Supporting Information). Nonetheless, in the top 14% variance group, prognosis was significantly different regarding both MFSs and OS (log‐rank test, *P* < 0.05) (Figure 1d; Figure S4f and i, Supporting Information). The silhouette plots also indicated good clustering of samples and genes in the top 14% variance group (Figure S4j, Supporting Information). To further assess the performance of the three models, receiver operating characteristic (ROC) analysis was performed. As shown in Figure S4k and l (Supporting Information, the top 14% variance model had the best areas under the curve (AUC) in both MFS and OS (AUC = 0.733 and 0.735, respectively). Thus, it was selected as the most robust consensus NMF clustering for the downstream analysis. The validity of clusters was visualized by Uniform Manifold Approximation and Projection (UMAP) analysis.

### Weighted Correlation Network Analysis (WGCNA) and Co‐Expressed Network Construction

The R software package “WGCNA”^[^ ^69^ ^]^ was used to identify the co‐expression modules. Genes with the top 25% variance were retained and subjected to clustering analysis. The co‐expression network fits a scale‐free network with β values ranging from 1 to 20. Also, a linear model was built by the adjacency of the nodes (log k) and the logarithm of the occurrence probability of the nodes^[^log(p(k))] with a correlation greater than 0.85. The nearest soft threshold was chosen for filtering the co‐expression module in order to ensure a scale‐free network. Next, the expression matrix was transformed to an adjacency matrix using “unsigned” type and thereafter to a topological matrix. In accordance with the topological overlap matrix (TOM), we used the mean linkage hierarchical clustering method to keep a minimum number of 30 genes in each module according to the hybrid dynamic cutting tree criterion. Afterwards, the eigengenes of each module was calculated, performed a cluster analysis of the modules, and then evaluated the relationship between the identified gene modules and the subtypes by calculating the Spearman's rank correlation coefficient. Hallmark gene sets, Reactome gene sets, and all GO gene sets were employed for functional annotations of the identified modules.

### Fusion Gene Detection

Screening for chimeric fusion transcripts was performed on raw fastq. files by two different bioinformatics pipelines, FusionCatcher^[^ ^70^ ^]^ (Version 1.10) and STAR‐Fusion^[^ ^71^ ^]^ (Version 1.2.0) with standard settings. A series of filtering processes to retain the putative true‐positive gene fusions as FusionCatcher and STAR‐Fusion both reported multiple fusion genes per sample was conceived, many of which had a high probability of being false‐positive.^[^ ^71^, ^72^ ^]^ Given the high noise due to FFPE samples and the low specificity of the fusion gene identification pipelines, a rigorous filtering process was performed in the FusionCatcher (Figures S4 and S18a, Supporting Information), excluding a). on a list of known false positives (banned; n = 979); b). fusion genes mapped to multiple genomic locations indicative of sequence homology (high common mapping reads; n = 1108); c). if one fusion gene partner had at least nine other fusion gene partners (promiscuous genes; n = 5459); d). fusion genes with less than three unique sequencing reads (low bioinformatics support; n = 1529); e). if both genes forming the fusion were adjacent or closely located in the genome (adjacent; n = 23); and f). the sequence of the fusion junction contained a highly repetitive region containing repeating short sequences or polyA/C/G/T (short repeats; n = 110), which were considered to be false positives and were removed before downstream analyses. Following the filtering process and subsequent inspection using Integrative Genomics Viewer (IGV), FusionCatcher identified 115 putative true fusion genes, including 96 in‐frame fusions, eight fusions with reciprocal reads, nine with supporting exonic reads, and two previously identified fusions (Mitelman/ChimerDB databases). STAR‐Fusion appeared to be less sensitive than FusionCatcher in the benchmark analysis, as the SS18::SSX fusion genes were detected only in 74 (81%) of the samples. After a series of filtering processes to exclude a). fusion genes with adjacent partners (adjacent genes; n = 108); b). fusions resulting from very local gene rearrangements (local rearrangements; n = 41); c). fusion transcripts with non‐canonical splice site sequences (non‐reference splice sites; n = 267); d). if one gene was fused with nine or more genes (“promiscuous” genes; n = 1), and e). genes that encoded procadherins (procadherins; n = 2), a total of 29 fusion genes were classified as putative true events (Figures S4 and S18b, Supporting Information). There were only five overlapping fusion events between the STAR‐Fusion and FusionCatcher whitelists (Figure S4, Supporting Information). Subsequently, all filtered fusion genes were manually inspected using the Integrative genomics viewer (IGV)^[^ ^73^ ^]^ and the R package “chimeraviz”^[^ ^74^ ^]^ to visualize the aligned reads. All the fusion gene information was shown in Table S2 (Supporting Information).

### Fusion Gene Verification using Sanger Sequencing

To validate the fusion genes detected by RNA‐seq, RNA was isolated from the same tumor with a separate punch biopsy using the QIAamp RNeasy FFPE Extraction Kit (Qiagen, Cat # 73 504). Sequencing primers were designed according to the full sequences and breakpoints using the output files from STAR‐fusion and FusionCatcher. Each fusion gene was verified using two sets of primer pairs. cDNA was synthesized using High‐Capacity RNA‐to‐cDNA Kit from Thermo Scientific (Cat # 4 387 406). After touch‐down PCR reactions (40 cycles), the amplification products were electrophoresed in a 5% agarose gel. The fusion genes were considered to pass the PCR validation if the size of the product corresponded to the expected size. The PCR products were isolated and purified by QIAquick PCR purification Kit (Qiagen, Cat # 28 104), and TA cloned into pCR4‐TOPO vector using TOPOTM Cloning TM Kit (Thermo Fisher, Cat # 450 071). The vectors were transformed into DH5a *E. coli* and cultured overnight on an LB agar plate, whereupon clones were selected and identified by PCR for DNA insertion. For positive clones, amplification in LB medium with 100ug/ml AMPr was conducted overnight. Finally, vectors were purified with the QIAprep Spin Miniprep KitBody Text (Qiagen, Cat #27 014) and Sanger sequenced with M13‐primers (Eurofins Genomics, Cologne, Germany) and manually analyzed using the 4Peaks Software (version 1.7.1, Mekentosj). All the whitelist fusion gene sequences and primers are shown in Table S3 (Supporting Information) (some whitelist fusion genes were excluded from PCR validation due to exhaustion of FFPE samples).

### Single Cell RNA Sequencing Pre‐Processing

The raw counts of scRNA‐seq data were processed with the Seurat package (v 4.10)^[^ ^75^ ^]^ for each individual sample. The cells with number of expressed genes < 300 and the gene expressed in less than 3 cells were filtered out. In addition, percentages of mitochondrial genes < 15%, ribosome genes > 1%, and hemoglobin <1% of total expressed genes were retained.

### Single Cell RNA Sequencing Data Analysis

For each sample, gene expression was denoted as a fraction of that gene and multiplied by 10 000, converted to the natural logarithm and normalized after adding 1 to avoid log 0 values. The batch effects were removed by the Harmony package (version 0.1.0)^[^ ^76^ ^]^ of R. We applied the “FindNeighbors” function in Saruat, using the 1st to 15th harmony dimensions and then looking for clusters at resolutions of 0.01, 0.05, 0.1, 0.2, 0.3, 0.5, 0.8 and 1. We identified clusters with resolution of 0.8 and calculated the UMAP coordinates using the same harmony dimensions for visualization. To annotate cell clusters, it was first filtered genes to include only those expressed in at least 25% of cells in at least one cluster at a given clustering resolution, and then “FindAllMarkers” function was executed with default parameters to find the cluster markers. It was assigned cells to different cell types according to the DEGs and canonical markers (Figure S5a and b, Supporting Information). The cellular markers were listed in Table S4 (Supporting Information). The malignant cells into three subsets was subdivided, with canonical mesenchymal cycling (*TOP2A* and *MKI67*), mesenchymal (*SNAI2*, *PDGFRA*, *ZEB2*, and *ZEB1*), and epithelial (*MUC1* and *EPCAM*) markers^[^ ^19^, ^77^ ^]^ to reflect the characteristics of subtypes, respectively. The other non‐malignant subsets were B cells, CAFs, CD4 cells, CD8 cells, endothelial, mast cells, myeloid, NK cells, and pericytes (Figure S5c, Supporting Information).

Single cell pseudotime trajectories were performed using the “Monocle2” package^[^ ^78^ ^]^ (v2.18.0) in R. The original UMI count‐scale gene‐cell matrix from the Seurat‐processed data was used as input. A newCellDataSet function was applied to create an object with expressionFamily = negbinomial.size and other default parameters. Only genes with mean expression ≥ 0.1 and genes with expression > 10 cells were selected for trajectory analysis. DEGs with q‐values <0.01 between cell groups were applied for dimensional reductions using the “reduceDimension” function with parameters reduction method = “DDRTree” and max components = 2. Cells were sorted and visualized with the function “plot_cell_trajectory”.

CellChat package (version 1.1.3) was used^[^ ^79^ ^]^ to explore the cell‐to‐cell interactions between malignant cells and immune cells and identify the role of ligands‐receptors in specific signaling pathways. The graphical visualization parameter was set at nPatterns = 5.

### DNA Sequencing

*Library preparation and sequencing*: A total of 20–250 ng DNA from each sample was used for library preparation with KAPA HyperPlus (Roche) with the following modifications: fragmentation with 12.5 min incubation, xGen Duplex Seq adapters (3‐4 nt unique molecular identifiers (UMI), 0.55 µM (for input amounts of 25–250 ng) or 0.15 µM (for input amounts of < 25 ng), Integrated DNA Technologies) were used for the ligation, and xGen Indexing primers (2 mM, with unique dual indices, Integrated DNA Technologies) were used for PCR amplification (5‐13 cycles depending on input amount of DNA). Target enrichment was performed in a multiplex fashion with a library amount of 375 ng (4‐plex). The libraries were hybridized to the capture probe using the Genomic Medicine Sweden mini panel (c387 genes GMS mini / GMCK v1) (Twist Bioscience) with the addition of Twist Universal Blockers and Blocking solution for 16 h (Table S5, Supporting Information). The post‐capture PCR was performed with xGen Library Amp Primer (0.5 mM, Integrated DNA Technologies) for ten cycles. Quality control was performed with the Quant‐iT dsDNA HS assay (Invitrogen) and TapeStation HS D1000 assay (Agilent). Sequencing was done on a NovaSeq 6000 (Illumina) using paired‐end 150 nt readout, aiming at 40 M read pairs per sample. Demultiplexing was done using the Illumina bcl2fastq Conversion Software v2.20.

### Target Sequencing Data Analysis‐Mutation Calling

To identify small tumor variants and single nucleotide variants (SNVs), we used the BALSAMIC pipeline v7.2.2^[^ ^80^ ^]^ to analyze each of the FASTQ files. First, quality control of fastq files was assessed using the FastQC software v 0.11.5,^[^ ^50^ ^]^ after which it was trimmed adapter sequences and low‐quality bases using the fastp v 0.20.0.^[^ ^81^ ^]^ Summarized quality results were created by MultiQC v 1.7.^[^ ^82^ ^]^ The trimmed reads were aligned to the reference genome (hg19) using BWA MEM v 0.7.15.^[^ ^83^ ^]^ The resulting SAM files were converted into BAM files and then sorted and indexed with Samtools v 1.6.^[^ ^84^ ^]^ The duplicate reads were marked using the Picard tools v2.17.0^[^ ^85^ ^]^ with MarkDuplicates and eliminated from the downstream analyses and promptly quality controlled using the CollectHsMetrics, CollectInsertSizeMetrics, and CollectAlignmentSummaryMetrics functionalities. For each sample, somatic mutations were called in paired variant calling mode using the VarDict v 2019.06.04^[^ ^86^ ^]^ and annotated using the Ensembl VEP v 99.1.^[^ ^87^ ^]^ For tumor mutational calling, all low‐quality variants were initially removed via a series of variant filtering using bcftools v 1.9.0,^[^ ^88^ ^]^ including Mean Mapping Quality (MQ) ≥ 40. Total read depth (DP) ≥ 100, Variant Depth (VD) ≥5.0, Allele frequency (AF) ≥ 0.01, and Maximum allele frequency across populations < 0.005 (1000Genomes Projects and ExAC). All passed variants were converted to Mutation Annotation Format (MAF) format using vcf2maf, with the annotation added by Ensembl VEP v 99.1.^[^ ^89^ ^]^ Subsequently, additional filtering was performed to reduce false‐positive calls and keep the somatic mutations: 1) variant allele frequency (VAF) > 10%; 2) Somatic != NULL; 3) Impact Variants = Moderate (non‐disruptive variants that might change protein effectiveness (e.g., missense mutations)) or high (for variants that are predicted to have a high impact (e.g., frameshift mutations)). Variants classified as putative functionally relevant and as of unknown/contradictory functional significance were selected, whereas neutral variants, alternations without representative gene/transcript, and polymorphisms (identified by dbSNP‐ GRCh37p13 version) were excluded from the downstream analyses by using the Molecular Tumor Board (MTB) Portal^[^ ^90^ ^]^ (accessed 11/2021). The latter was a clinical decision support tool that provides a general interpretation of a given list of cancer gene variants that combines the latest results from clinical and preclinical studies, true biological hypotheses, and bioinformatics calculations to classify a variant as biologically relevant and to evaluate the functional and predictive relevance of genomic alterations. The TMB of each tumor was calculated with the “tmb” function of the R package “maftools”.^[^ ^48^ ^]^

CNVkit v 0.9.4a0^[^ ^91^ ^]^ was used with default parameters on paired tumor‐normal sequencing data. The log2 copy ratio in the cnr.file from the CNVkit program was adjusted to represent the length of the bin, and copy number segments from the given coverage table using default function circular binary segmentation (CBS).

### HiRIEF‐nanoLC‐MS/MS based Proteomics

A total of 14 fresh frozen SS samples were dissolved in 200 µl lysis buffer (4% SDS, 50 mM Hepes, 1 mM DTT, pH 7.4) and the total protein amount was estimated (DC kit, BioRad). Samples were heated and sonicated then prepared for mass spectrometry analysis using a modified version of the SP3 protein clean‐up and a digestion protocol,^[^ ^92^ ^]^ where proteins were digested by LysC and trypsin (sequencing grade modified, Pierce). In brief, ≈100 µg protein from each sample was alkylated with 4 mM chloroacetamide, sera‐Mag SP3 bead mix (20 µl) was added to the protein sample together with 100% acetonitrile to a final concentration of 60%, and the mix was incubated under rotation at room temperature for 20 min. The mix was then placed on a magnetic rack and the supernatant was discarded, followed by two washes with 70% ethanol and one with 100% acetonitrile. The beads‐protein mixture was reconstituted in 100 µl LysC buffer (0.5 M Urea, 50 mM HEPES pH: 7.6 and 1:50 enzyme (LysC) to protein ratio) and incubated overnight. Finally, trypsin was added in 1:50 enzyme to protein ratio in 100 µl 50 mM HEPES pH 7.6 and incubated overnight. The peptides were eluted from the mixture after placing the mixture on a magnetic rack, followed by peptide concentration measurement (DC kit, BioRad). The samples were then pH adjusted using TEAB pH 8.5 (100 mM final conc.), 40 µg of peptides from each sample were labelled with isobaric TMT‐tags (TMTpro 16 plex reagent) according to the manufacturer's protocol (Thermo Scientific), and 320 µg of peptides separated by immobilized pH gradient – isoelectric focusing (IPG‐IEF) on 3–10 strip and 3.7–4.9 strip as described previously.^[^ ^93^ ^]^

Labelling efficiency was determined by LC‐MS/MS before pooling of the samples. For the sample clean‐up step, a solid phase extraction (SPE strata‐X‐C, Phenomenex) of TMT labelled and pooled peptides was performed and purified samples were dried in a SpeedVac. An aliquot of ≈10 µg was suspended in LC mobile phase A and 2 µg was injected on the LC‐MS/MS system. Online LC‐MS was performed as previously described^[^ ^93^ ^]^ using a Dionex UltiMate 3000 RSLCnano System coupled to a Q‐Exactive‐HF mass spectrometer (Thermo Scientific). Each of the 72 fractions in the plate wells was dissolved in 20 µL solvent A and 10 µL were injected. Samples were trapped on a C18 guard‐desalting column (Acclaim PepMap 100, 75 µm x 2 cm, nanoViper, C18, 5 µm, 100 Å), and separated on a 50 cm long C18 column (Easy spray PepMap RSLC, C18, 2 µm, 100 Å, 75 µm x 50 cm). The nano capillary solvent A was 95% water, 5% DMSO, 0.1% formic acid; and solvent B was 5% water, 5% DMSO, 95% acetonitrile, 0.1% formic acid. At a constant flow of 0.25 µl min^−1^, the curved gradient went from 6%–10% B up to 40% B in each fraction in a dynamic range of gradient length, followed by a steep increase to 100% B in 5 min. FTMS master scans with 60 000 resolution (and mass range 300–1500 m z^−1^) were followed by data‐dependent MS/MS (30 000 resolution) on the top 5 ions using higher energy collision dissociation (HCD) at 30% normalized collision energy. Precursors were isolated with a 2 m z^−1^ window. Automatic gain control (AGC) targets were 1e6 for MS1 and 1e5 for MS2. Maximum injection times were 100 ms for MS1 and 100 ms for MS2. The entire duty cycle lasted ≈2.5 s. Dynamic exclusion was used with 30 s duration. Precursors with unassigned charge state or charge state 1 were excluded. An underfill ratio of 1% was used.

Orbitrap raw MS/MS files were converted to mzML format using msConvert from the ProteoWizard tool suite.^[^ ^94^ ^]^ Spectra were then searched using MSGF+ (v10072)^[^ ^95^ ^]^ and Percolator (v2.08).^[^ ^96^ ^]^ All searches were done against the human protein subset of Ensembl (ENS104) in the Nextflow platform (https://github.com/lehtiolab/ddamsproteomics, vs1.5).

Pairwise subtype differences at the protein level were estimated with DEqMS (v1.12.1).^[^ ^97^ ^]^ Enrichment analysis was performed on the sorted log2 fold changes with fgsea (v1.20.0)^[^ ^98^ ^]^ using MSigDB Reactome canonical pathways v7.4^[^ ^59^ ^]^ and significant pathways were called at an FDR < 0.01.

### Immunohistochemistry

For the validation of protein expression at the histological level, we selected the Rabbit monoclonal anti‐KRT8 (clone 2174R, ab234348 from Abcam at 1 µg ml^−1^ dilution). To determine the optimal antibody concentration, we initially used anonymized normal human tissue controls (colon, testis, and small bowel), as well as two selected synovial sarcoma cases the tumors representing high and low gene expression of the target genes.

A total of 21 cases were chosen for immunohistochemistry (IHC) based on relative gene expression differences of KRT8. From formalin‐fixed paraffin‐embedded (FFPE) blocks, we obtained 4 µm thick sections, which were then deparaffinized. For all antibodies, heat‐induced antigen retrieval was performed using a 20 min incubation with citrate buffer (pH 6.0) and microwave heating. Blocking was carried out using 10% BSA. Incubation with primary antibodies occurred overnight at 4 degrees Celsius. Following secondary antibody incubation, sections were developed using 3,3′‐diaminobenzidine (DAB) and counterstained with hematoxylin.

Using brightfield microscopy, we reviewed the entire slide for each case. Antibody staining patterns were evaluated and quantified by two authors in consensus (CH and FHdF). After reviewing the staining patterns, all cases were scored relatively using arbitrary ordinal categories: “Negative” (0), “Weak” (1) – indicating weak staining in at least 25% of tumor cells, or “Moderate” (2) – indicating moderate staining in at least 25% of tumor cells. No cases exhibited “strong” immunoreactivity for any antigen. All photomicrographs were captured at a magnification of x400.

### Statistics

The unpaired Student's t‐test was used to analyze the comparison between two continuous variables and a normally distributed variable. Non‐normally distributed variables were analyzed with the Mann‐Whitney U test. The Kaplan‐Meier method in the R package “survminer” and “survival” was used for survival analysis with the log‐rank test for comparisons between groups. In order to compare three or more groups, ANOVA and Kruskal‐Wallis tests were performed on parametric and non‐parametric variables respectively. Correlations of rank‐ordered values were assessed using the Spearman rank correlation test. Statistical analysis was conducted in the R version 4.0.3 (R Foundation for Statistical Computing, Vienna, Austria).

### Ethics Approval and Consent to Participate

The study and collection of patients' samples were approved by the local ethics committee (The Regional Ethics Committee in Stockholm), reference number 2013 1979‐31. All patients had given oral and written consent prior to sample collection. The study was performed in agreement with the Declaration of Helsinki.

## Conflict of Interest

The authors declare no conflict of interest.

## Author Contributions

Y.C. and F.H., conceived the project, designed the study. Y.C., I.R.L., I.S., H.J., W.‐K.H, J.L., X.L., and F.H. interpreted results. F.H. and O.L. obtained funding for the study. P.T., A.C.H., A.P., Y.Z., and F.H. consented patients for the study, collected synovial sarcoma samples, and provide the clinical data. Y.Z. and F.H. performed the pathological evaluations and tissue biopsies. Y.C. performed transcriptomic and genomic analyses. I.S. and Y.C. performed the proteomics analyses. J.Z. provided support for single‐cell RNA analyses. Y.C. and Y.L. performed RNA and DNA sample preparations. Y.C., X.C., and H.J. performed proteomics sample preparation, MS data generation and searching. X.L. and Y.L. performed the experimental validations of the fusion genes. Y.S. and M.E. performed the multiplexing and digital image analysis. Y.C., X.L., and F.H. wrote the manuscript with feedback from all authors.

## Supporting information

Supporting Information

Supporting Tables

## Data Availability

The raw and processed RNA‐seq data can be accessed through the Gene Expression Omnibus (GEO) https://www.ncbi.nlm.nih.gov/geo/ under accession number GSE271517. The processed panel sequencing data have been deposited at the European Genome‐Phenome Archive (EGA) under accession number EGAS50000000522. MS proteomics data for DDA and DIA analyses have been deposited in the ProteomeXchange Consortium via the Proteomics Identification Database (PRIDE) partner repository with the dataset identifiers PXD053789. Previously published gene expression data re‐analyzed in this study are available under accession codes GSE40021 and GSE131309, with chromosomal arm‐level variation data available under accession code GSE54183. All these datasets, including relevant clinical information, were retrieved from the NCBI GEO database. Additionally, the Sarsoma_MSKCC_2022 cohort was used to validate somatic copy number alterations (SCNAs) and single nucleotide variants (SNVs) in our genomic analysis, accessible through cBioPortal https://www.cbioportal.org/study/summary?id=sarcoma_mskcc_2022. For pan‐cancer analysis, the uniformly normalized pan‐cancer dataset, TCGA Pan‐Cancer (PANCAN, N=10535, G=60499), was downloaded from the UCSC Xena platform https://xenabrowser.net/. Relevant outputs of data analysis are provided in the Supplementary Data. All other data supporting the findings of this study are available from the corresponding author upon reasonable request.
