# Supplementary material for: Molecular Profiling Defines Three Subtypes of Synovial Sarcoma
Source: Adv Sci (Weinh). 2024 Sep 10;11(41):2404510. doi: 10.1002/advs.202404510 (PMC11892499; doi:10.1002/advs.202404510)
Supplement: Supplementary file 1 — Supporting Information [file ADVS-11-2404510-s001.pdf]

ADVANCED SCIENCE
Open Access

Supporting Information

for *Adv. Sci.*, DOI 10.1002/advs.202404510

Molecular Profiling Defines Three Subtypes of Synovial Sarcoma

Yi Chen, Yanhong Su, Xiaofang Cao, Ioannis Siavelis, Isabelle Rose Leo, Jianming Zeng, Panagiotis Tsagkozis, Asle C. Hesla, Andri Papakonstantinou, Xiao Liu, Wen-Kuan Huang, Binbin Zhao, Cecilia Haglund, Monika Ehnman, Henrik Johansson, Yingbo Lin, Janne Lehtiö, Yifan Zhang, Olle Larsson, Xuexin Li* and Felix Haglund de Flon**

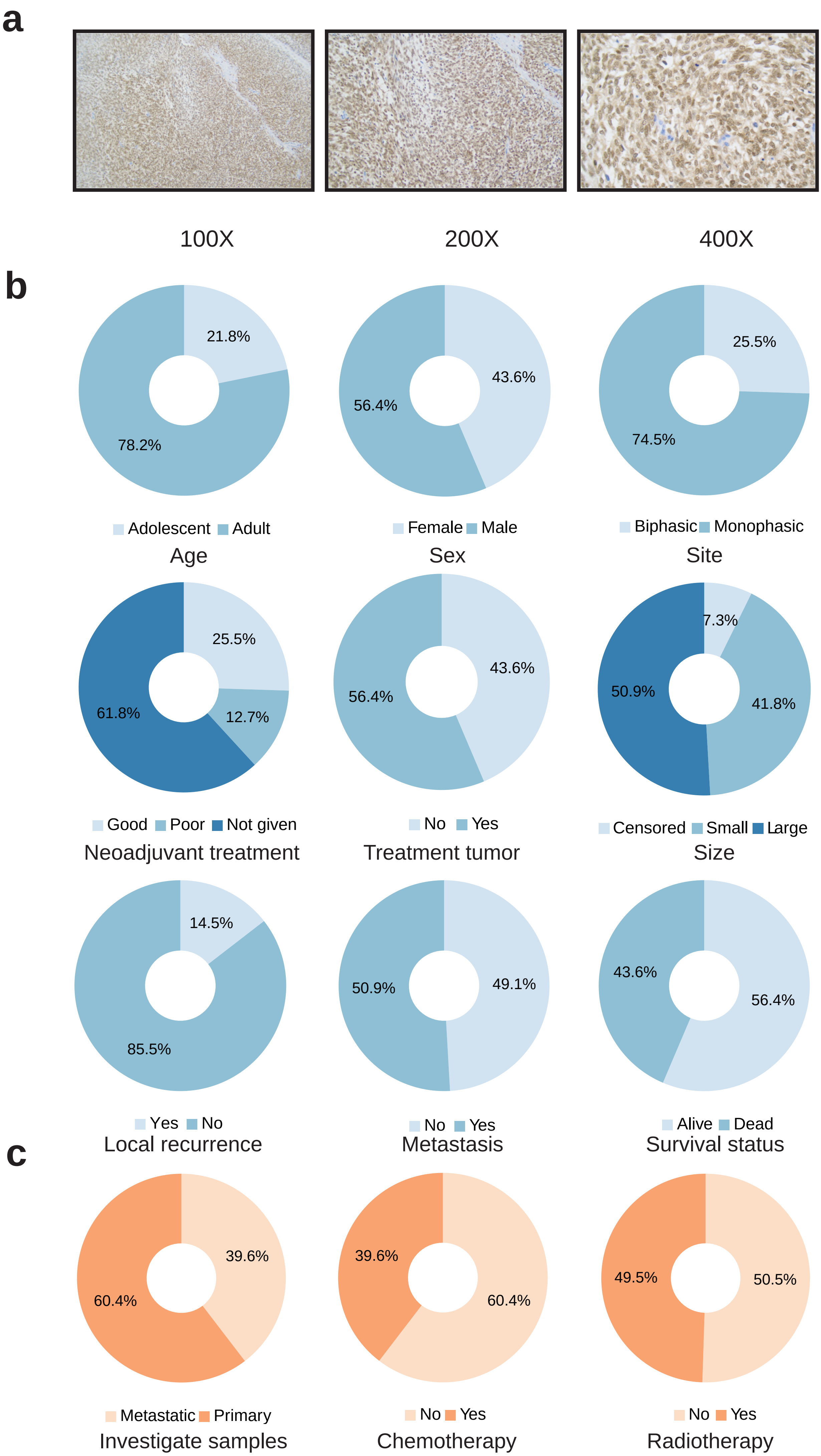
a
100X
200X
400X
b
21.8%
78.2%
Adolescent
Adult
Age
43.6%
56.4%
Female
Male
Sex
25.5%
74.5%
Biphasic
Monophasic
Site
25.5%
12.7%
61.8%
Good
Poor
Not given
Neoadjuvant treatment
43.6%
56.4%
No
Yes
Treatment tumor
7.3%
41.8%
50.9%
Censored
Small
Large
Size
14.5%
85.5%
Yes
No
Local recurrence
49.1%
50.9%
No
Yes
Metastasis
56.4%
43.6%
Alive
Dead
Survival status
c
39.6%
60.4%
Metastatic
Primary
Investigate samples
39.6%
60.4%
No
Yes
Chemotherapy
50.5%
49.5%
No
Yes
Radiotherapy

Figure S1: (a) SS18-SSX immunoreactivity at 100 ×, 200 ×, and 400 × magnifications. **(b-c).** Summary of clinical characteristics of **(b)** 55 synovial sarcoma patients and **(c)** 91 patients.

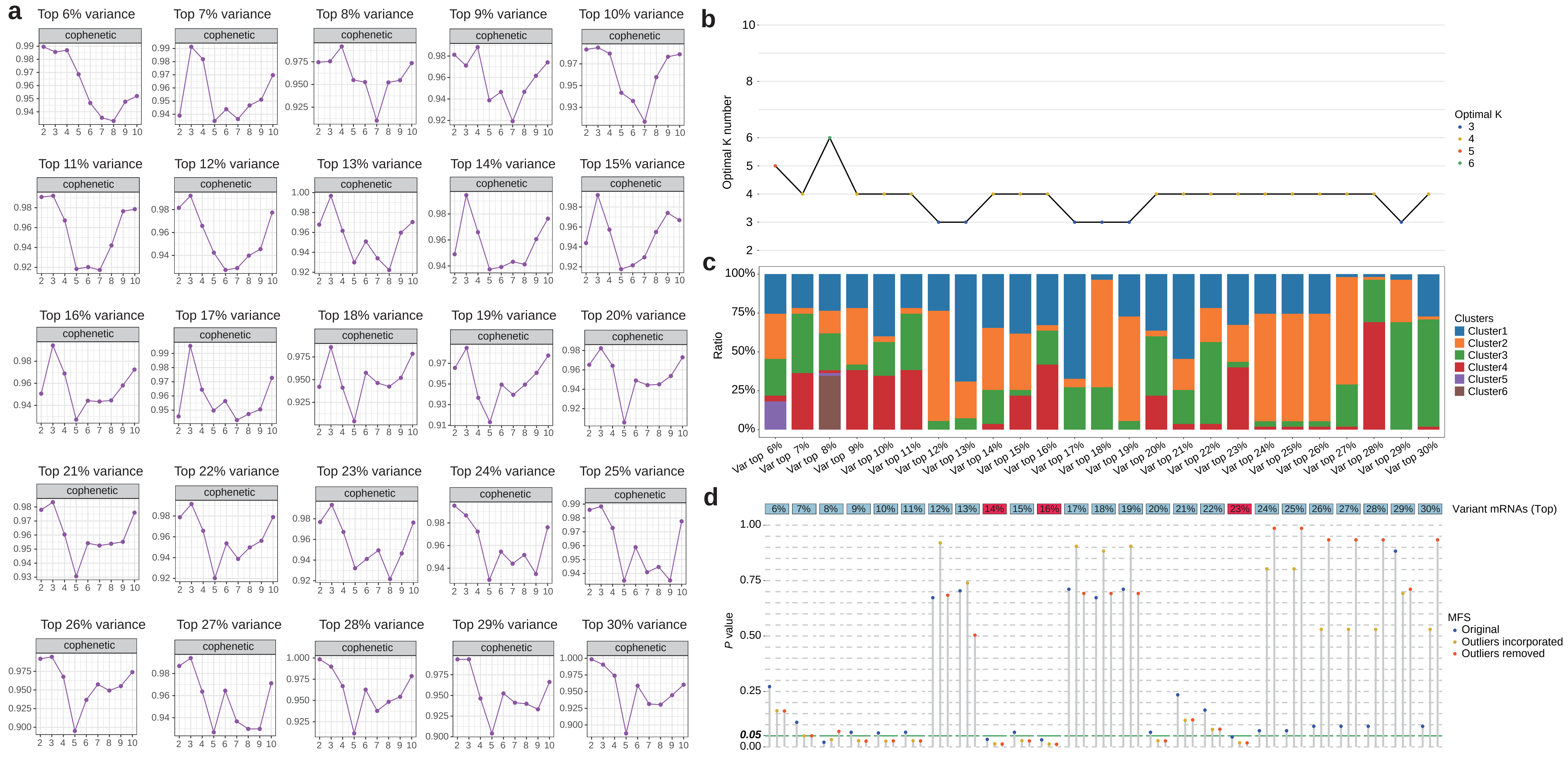

a
Top 6% variance
Top 7% variance
Top 8% variance
Top 9% variance
Top 10% variance
Top 11% variance
Top 12% variance
Top 13% variance
Top 14% variance
Top 15% variance
Top 16% variance
Top 17% variance
Top 18% variance
Top 19% variance
Top 20% variance
Top 21% variance
Top 22% variance
Top 23% variance
Top 24% variance
Top 25% variance
Top 26% variance
Top 27% variance
Top 28% variance
Top 29% variance
Top 30% variance
cophenetic
b
Optimal K number
Optimal K
3
4
5
6
c
Ratio
Clusters
Cluster1
Cluster2
Cluster3
Cluster4
Cluster5
Cluster6
Var top 6%
Var top 7%
Var top 8%
Var top 9%
Var top 10%
Var top 11%
Var top 12%
Var top 13%
Var top 14%
Var top 15%
Var top 16%
Var top 17%
Var top 18%
Var top 19%
Var top 20%
Var top 21%
Var top 22%
Var top 23%
Var top 24%
Var top 25%
Var top 26%
Var top 27%
Var top 28%
Var top 29%
Var top 30%
d
Variant mRNAs (Top)
P value
MFS
Original
Outliers incorporated
Outliers removed

Figure S2: (a). Cophenetic correlation plots from top 6% to top 30% variance in mRNAs. **(b).** NMF clustering of the distribution of the optimal K numbers from the top 6% to the top 30% variant mRNAs. **(c).** NMF clustering of the distribution of the number of patients from the top 6% to the top 30% variant mRNAs. **(d).** NMF clustering of the distribution of the MFS values (original, outliers incorporated, outliers removed) from the top 6% to the top 30% variant mRNAs.

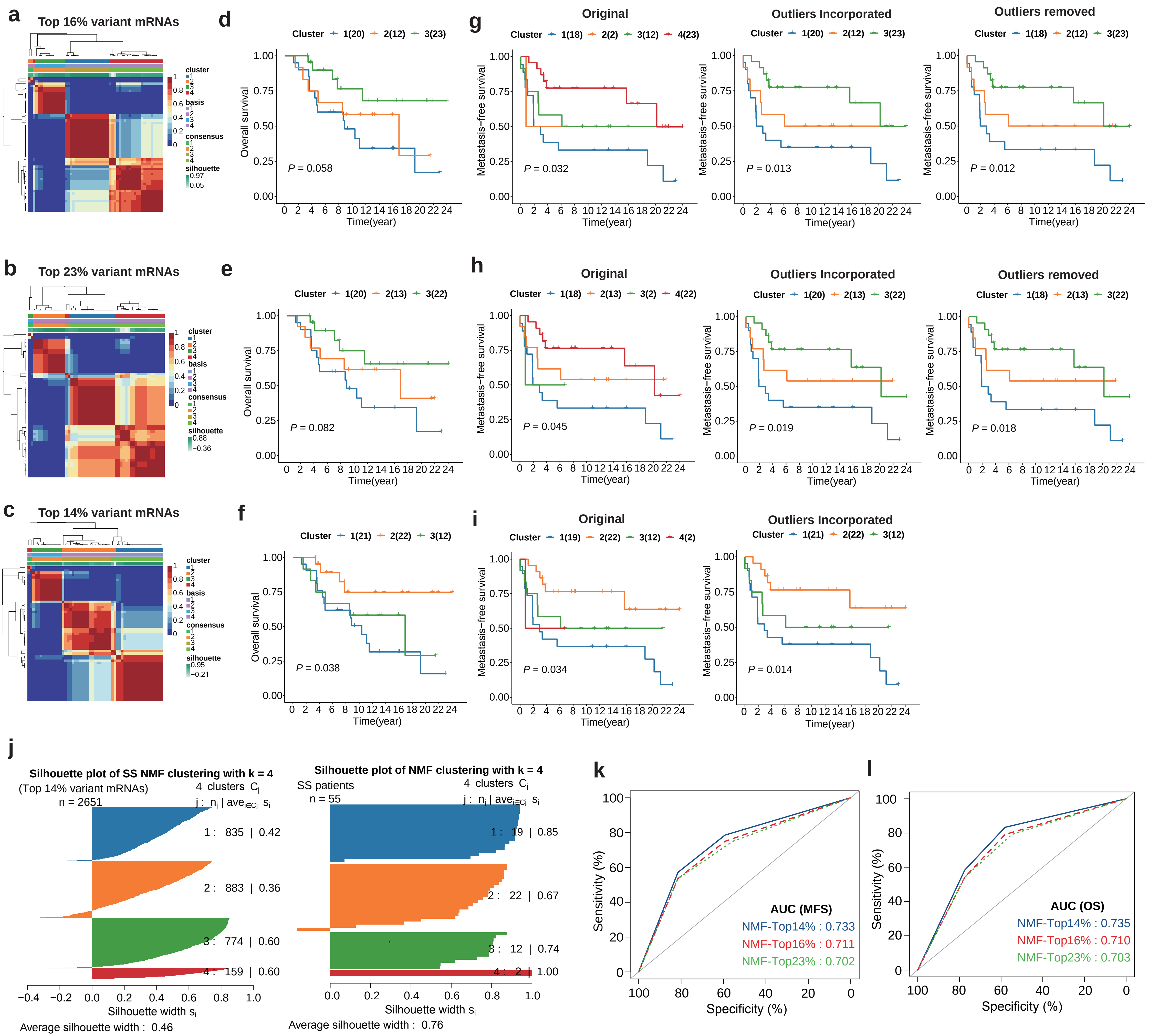
a
Top 16% variant mRNAs
b
Top 23% variant mRNAs
c
Top 14% variant mRNAs
cluster
basis
consensus
silhouette
d
Cluster 1(20) 2(12) 3(23)
Overall survival
P = 0.058
Time(year)
e
Cluster 1(20) 2(13) 3(22)
P = 0.082
f
Cluster 1(21) 2(22) 3(12)
P = 0.038
g
Original
Cluster 1(18) 2(2) 3(12) 4(23)
Metastasis−free survival
P = 0.032
Outliers Incorporated
Cluster 1(20) 2(12) 3(23)
P = 0.013
Outliers removed
Cluster 1(18) 2(12) 3(23)
P = 0.012
h
Original
Cluster 1(18) 2(13) 3(2) 4(22)
P = 0.045
Outliers Incorporated
Cluster 1(20) 2(13) 3(22)
P = 0.019
Outliers removed
Cluster 1(18) 2(13) 3(22)
P = 0.018
i
Original
Cluster 1(19) 2(22) 3(12) 4(2)
P = 0.034
Outliers Incorporated
Cluster 1(21) 2(22) 3(12)
P = 0.014
j
Silhouette plot of SS NMF clustering with k = 4
(Top 14% variant mRNAs)
n = 2651
4 clusters Cj
j : nj | ave i∈Cj si
1 : 835 | 0.42
2 : 883 | 0.36
3 : 774 | 0.60
4 : 159 | 0.60
Silhouette width si
Average silhouette width : 0.46
Silhouette plot of NMF clustering with k = 4
SS patients
n = 55
1 : 19 | 0.85
2 : 22 | 0.67
3 : 12 | 0.74
4 : 2 | 1.00
Average silhouette width : 0.76
k
Sensitivity (%)
Specificity (%)
AUC (MFS)
NMF-Top14% : 0.733
NMF-Top16% : 0.711
NMF-Top23% : 0.702
l
AUC (OS)
NMF-Top14% : 0.735
NMF-Top16% : 0.710
NMF-Top23% : 0.703

Figure S3: (a-c). Consensus matrix of NMF clustering at top 16%, 23%, and 14% mRNAs variance. **(d-f).** Kaplan-Meier curve of overall survival in synovial sarcoma patients with clusters outliers incorporated at top 16%, 23%, and 14% mRNAs variance (Log-rank test, P = 0.058, 0.082, and 0.038, respectively). **(g).** Kaplan-Meier curve of clusters of metastasis-free survival in synovial sarcoma patients (original, outliers incorporated, outliers removed) at top 16% mRNAs variance (Log-rank test, P = 0.014, 0.013, and 0.012 respectively). **(h).** Kaplan-Meier curve of clusters of metastasis-free survival in synovial sarcoma patients (original, outliers incorporated, outliers removed) at top 23% mRNAs variance (Log-rank test, P = 0.045, 0.019, and 0.018 respectively). **(i).** Kaplan-Meier curve of clusters of metastasis-free survival in synovial sarcoma patients (original, outliers incorporated) at top 14% mRNAs variance (Log-rank test, P = 0.034, and 0.014, respectively). **(j).** Silhouette plot of NMF clustering of genes and patients at top 14% mRNAs variance. **(k-l).** ROC curves of the prediction performances of top 14%, 16%, and 23% variances in MFS and OS, respectively.

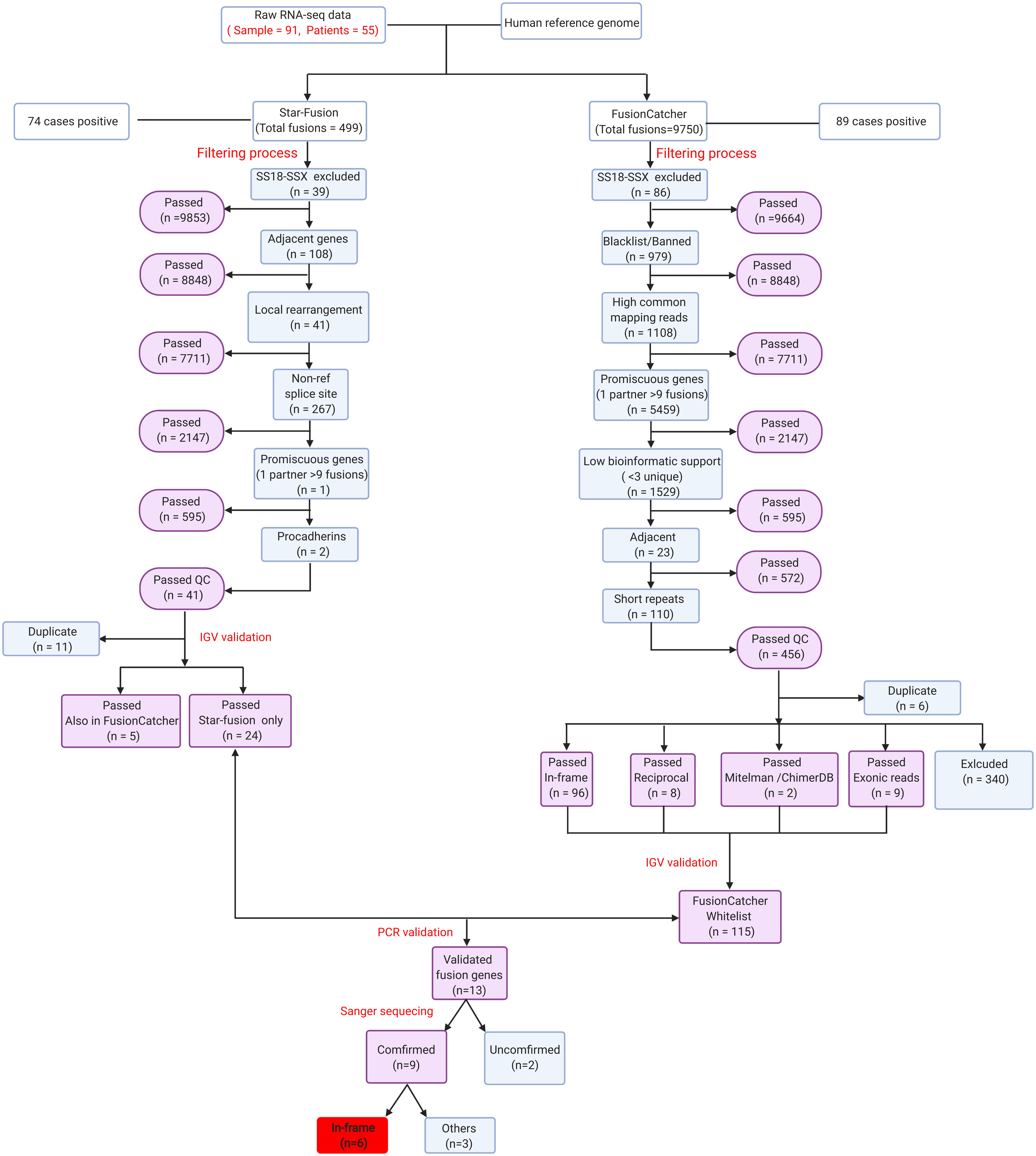
Raw RNA-seq data
(Sample = 91, Patients = 55)
Human reference genome
Star-Fusion
(Total fusions = 499)
74 cases positive
FusionCatcher
(Total fusions=9750)
89 cases positive
Filtering process
SS18-SSX excluded
(n = 39)
Passed
(n =9853)
Adjacent genes
(n = 108)
Passed
(n = 8848)
Local rearrangement
(n = 41)
Passed
(n = 7711)
Non-ref
splice site
(n = 267)
Passed
(n = 2147)
Promiscuous genes
(1 partner >9 fusions)
(n = 1)
Passed
(n = 595)
Procadherins
(n = 2)
Passed QC
(n = 41)
Duplicate
(n = 11)
IGV validation
Passed
Also in FusionCatcher
(n = 5)
Passed
Star-fusion only
(n = 24)
Filtering process
SS18-SSX excluded
(n = 86)
Passed
(n =9664)
Blacklist/Banned
(n = 979)
Passed
(n = 8848)
High common
mapping reads
(n = 1108)
Passed
(n = 7711)
Promiscuous genes
(1 partner >9 fusions)
(n = 5459)
Passed
(n = 2147)
Low bioinformatic support
(<3 unique)
(n = 1529)
Passed
(n = 595)
Adjacent
(n = 23)
Passed
(n = 572)
Short repeats
(n = 110)
Passed QC
(n = 456)
Duplicate
(n = 6)
Passed
In-frame
(n = 96)
Passed
Reciprocal
(n = 8)
Passed
Mitelman /ChimerDB
(n = 2)
Passed
Exonic reads
(n = 9)
Exlcuded
(n = 340)
IGV validation
FusionCatcher
Whitelist
(n = 115)
PCR validation
Validated
fusion genes
(n=13)
Sanger sequecing
Comfirmed
(n=9)
Uncomfirmed
(n=2)
In-frame
(n=6)
Others
(n=3)

Figure S4: Workflow of the fusion gene filtering process.

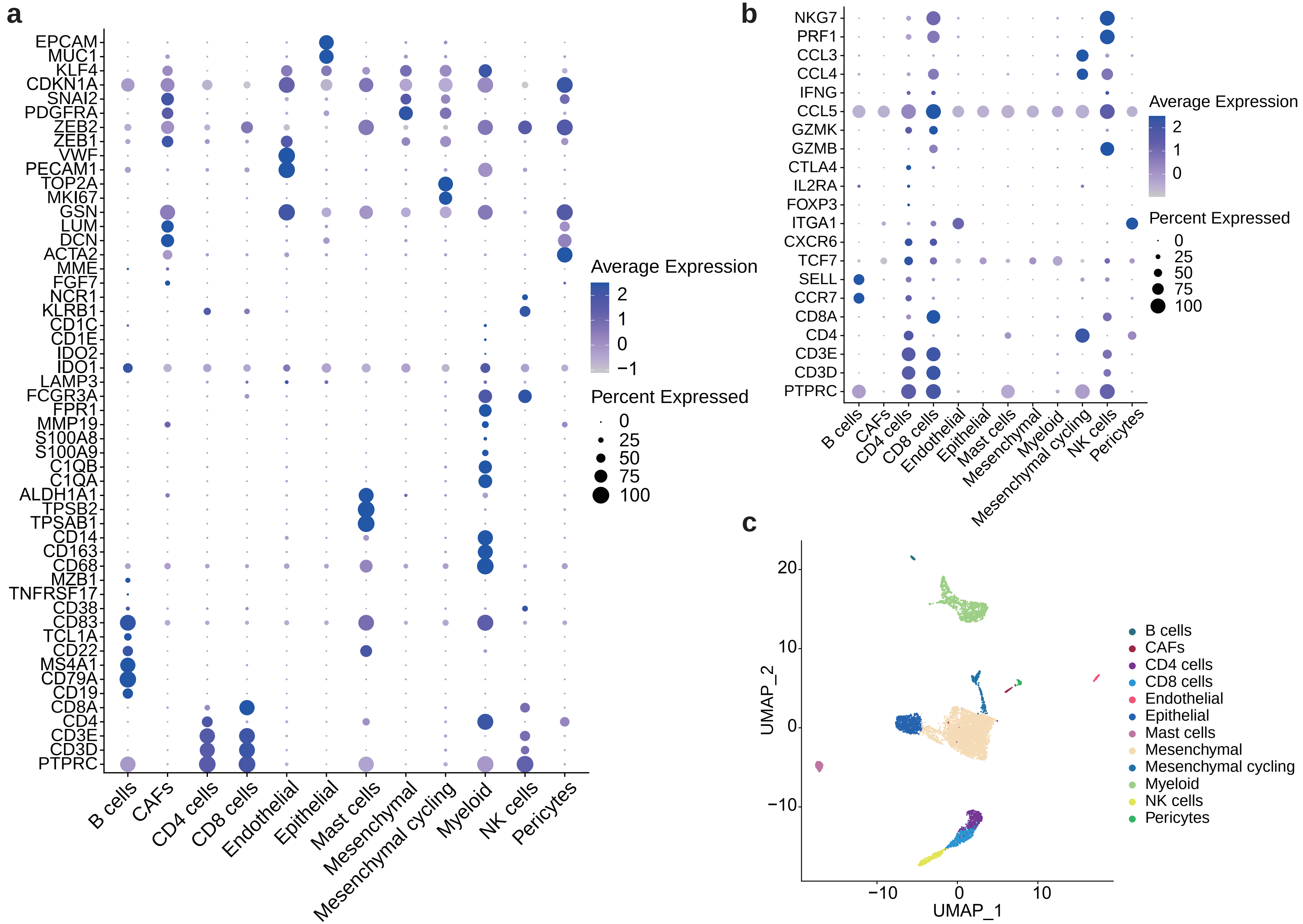
a
EPCAM
MUC1
KLF4
CDKN1A
SNAI2
PDGFRA
ZEB2
ZEB1
VWF
PECAM1
TOP2A
MKI67
GSN
LUM
DCN
ACTA2
MME
FGF7
NCR1
KLRB1
CD1C
CD1E
IDO2
IDO1
LAMP3
FCGR3A
FPR1
MMP19
S100A8
S100A9
C1QB
C1QA
ALDH1A1
TPSB2
TPSAB1
CD14
CD163
CD68
MZB1
TNFRSF17
CD38
CD83
TCL1A
CD22
MS4A1
CD79A
CD19
CD8A
CD4
CD3E
CD3D
PTPRC
B cells
CAFs
CD4 cells
CD8 cells
Endothelial
Epithelial
Mast cells
Mesenchymal
Mesenchymal cycling
Myeloid
NK cells
Pericytes
Average Expression
2
1
0
−1
Percent Expressed
0
25
50
75
100
b
NKG7
PRF1
CCL3
CCL4
IFNG
CCL5
GZMK
GZMB
CTLA4
IL2RA
FOXP3
ITGA1
CXCR6
TCF7
SELL
CCR7
CD8A
CD4
CD3E
CD3D
PTPRC
B cells
CAFs
CD4 cells
CD8 cells
Endothelial
Epithelial
Mast cells
Mesenchymal
Myeloid
Mesenchymal cycling
NK cells
Pericytes
Average Expression
2
1
0
Percent Expressed
0
25
50
75
100
c
20
10
0
−10
UMAP_2
−10
0
10
UMAP_1
B cells
CAFs
CD4 cells
CD8 cells
Endothelial
Epithelial
Mast cells
Mesenchymal
Mesenchymal cycling
Myeloid
NK cells
Pericytes

Figure S5: (a-b). Dot plots showing the **(a)** cell markers (except T cell markers) and **(b)** T cell markers expressions across the 12 cellular clusters. The size of dots represents the proportion of cells expressing the particular marker, and the spectrum of color indicates the mean expression levels of the markers. **(c).** UMAP plot of scRNA-seq profiles all SS patients, colored by cell types.

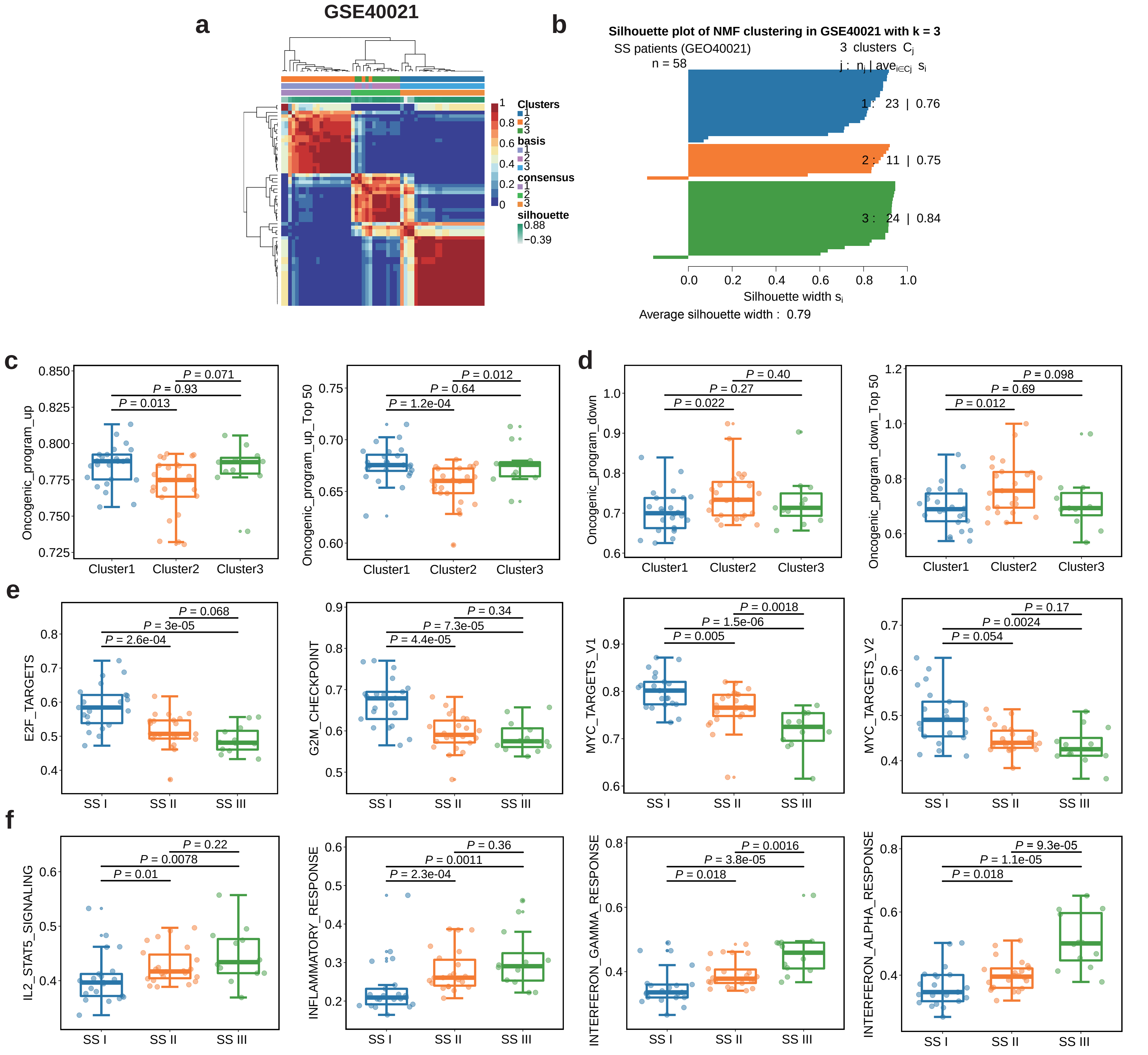

a
GSE40021
Clusters
basis
consensus
silhouette
0.88
−0.39
b
Silhouette plot of NMF clustering in GSE40021 with k = 3
SS patients (GEO40021)
n = 58
3 clusters C_j
j : n_j | $ave_{i \in Cj}$ s_i
1 : 23 | 0.76
2 : 11 | 0.75
3 : 24 | 0.84
Silhouette width s_i
Average silhouette width : 0.79
c
Oncogenic_program_up
Oncogenic_program_up_Top 50
Cluster1
Cluster2
Cluster3
P = 0.071
P = 0.93
P = 0.013
P = 0.012
P = 0.64
P = 1.2e-04
d
Oncogenic_program_down
Oncogenic_program_down_Top 50
P = 0.40
P = 0.27
P = 0.022
P = 0.098
P = 0.69
P = 0.012
e
E2F_TARGETS
G2M_CHECKPOINT
MYC_TARGETS_V1
MYC_TARGETS_V2
SS I
SS II
SS III
P = 0.068
P = 3e-05
P = 2.6e-04
P = 0.34
P = 7.3e-05
P = 4.4e-05
P = 0.0018
P = 1.5e-06
P = 0.005
P = 0.17
P = 0.0024
P = 0.054
f
IL2_STAT5_SIGNALING
INFLAMMATORY_RESPONSE
INTERFERON_GAMMA_RESPONSE
INTERFERON_ALPHA_RESPONSE
P = 0.22
P = 0.0078
P = 0.01
P = 0.36
P = 0.0011
P = 2.3e-04
P = 0.0016
P = 3.8e-05
P = 0.018
P = 9.3e-05
P = 1.1e-05
P = 0.018

Figure S6: (a). Consensus matrix of NMF clustering at top 14% mRNAs variance applied at GSE40021 validation cohort. **(b).** Silhouette plot of NMF clustering of patients at top 14% mRNAs variance applied at GSE40021 validation cohort. **(c-d).** Distributions of the ssGSEA score of the *core oncogenic program* **(d)** upregulated genes and **(e)** downregulated genes in the three SS subtypes at GSE40021. Middle line: median; box edges: 25th and 75th percentiles. Mann-Whitney U test. **(e-f).** Distributions of the ssGSEA score of **(e)** four significant proliferative and **(f)** immune-related hallmarks in the three SS subtypes. Middle line: median; box edges: 25th and 75th percentiles. Mann-Whitney U test.

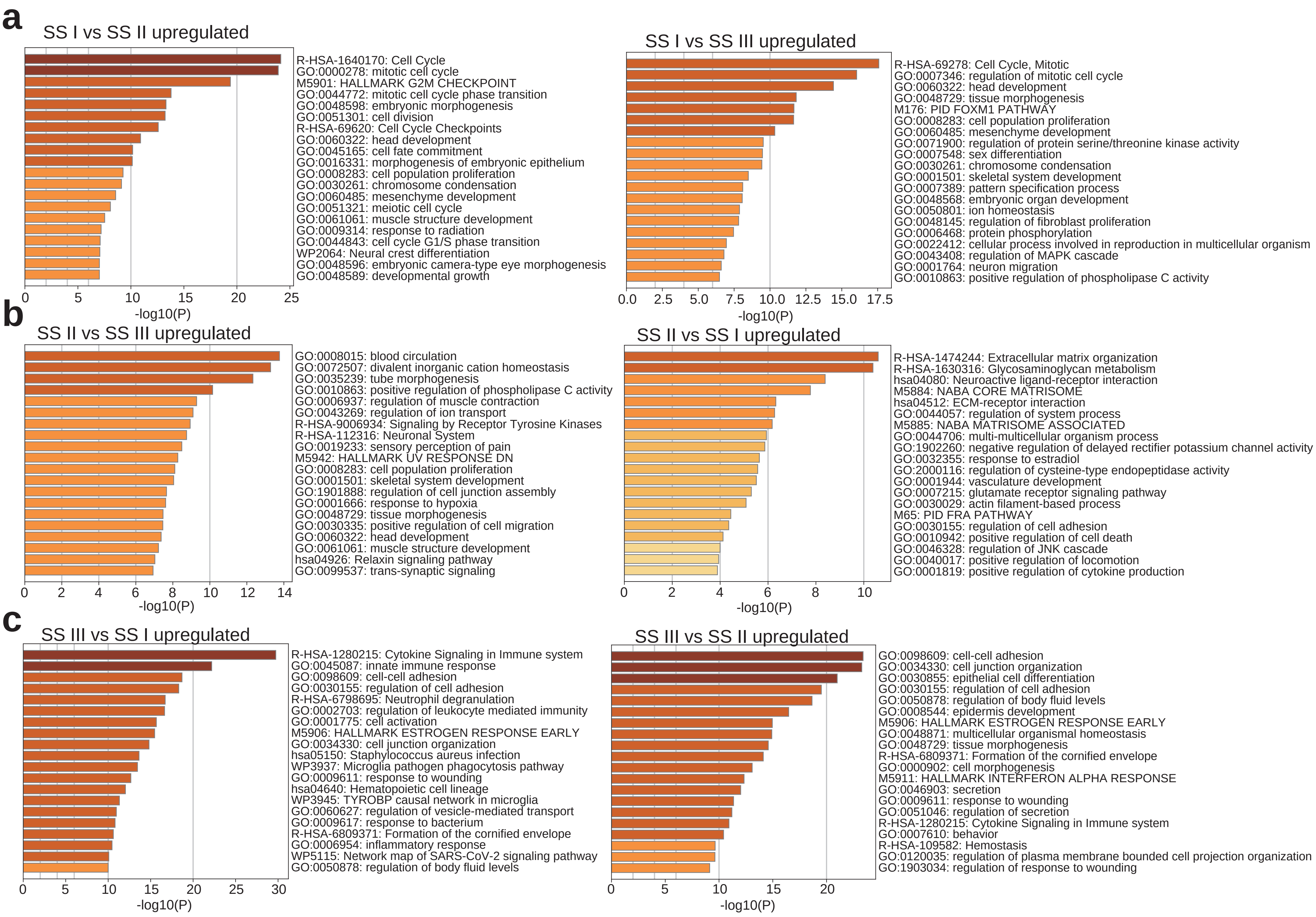

a
SS I vs SS II upregulated
R-HSA-1640170: Cell Cycle
GO:0000278: mitotic cell cycle
M5901: HALLMARK G2M CHECKPOINT
GO:0044772: mitotic cell cycle phase transition
GO:0048598: embryonic morphogenesis
GO:0051301: cell division
R-HSA-69620: Cell Cycle Checkpoints
GO:0060322: head development
GO:0045165: cell fate commitment
GO:0016331: morphogenesis of embryonic epithelium
GO:0008283: cell population proliferation
GO:0030261: chromosome condensation
GO:0060485: mesenchyme development
GO:0051321: meiotic cell cycle
GO:0061061: muscle structure development
GO:0009314: response to radiation
GO:0044843: cell cycle G1/S phase transition
WP2064: Neural crest differentiation
GO:0048596: embryonic camera-type eye morphogenesis
GO:0048589: developmental growth
-log10(P)
SS I vs SS III upregulated
R-HSA-69278: Cell Cycle, Mitotic
GO:0007346: regulation of mitotic cell cycle
GO:0060322: head development
GO:0048729: tissue morphogenesis
M176: PID FOXM1 PATHWAY
GO:0008283: cell population proliferation
GO:0060485: mesenchyme development
GO:0071900: regulation of protein serine/threonine kinase activity
GO:0007548: sex differentiation
GO:0030261: chromosome condensation
GO:0001501: skeletal system development
GO:0007389: pattern specification process
GO:0048568: embryonic organ development
GO:0050801: ion homeostasis
GO:0048145: regulation of fibroblast proliferation
GO:0006468: protein phosphorylation
GO:0022412: cellular process involved in reproduction in multicellular organism
GO:0043408: regulation of MAPK cascade
GO:0001764: neuron migration
GO:0010863: positive regulation of phospholipase C activity
-log10(P)
b
SS II vs SS III upregulated
GO:0008015: blood circulation
GO:0072507: divalent inorganic cation homeostasis
GO:0035239: tube morphogenesis
GO:0010863: positive regulation of phospholipase C activity
GO:0006937: regulation of muscle contraction
GO:0043269: regulation of ion transport
R-HSA-9006934: Signaling by Receptor Tyrosine Kinases
R-HSA-112316: Neuronal System
GO:0019233: sensory perception of pain
M5942: HALLMARK UV RESPONSE DN
GO:0008283: cell population proliferation
GO:0001501: skeletal system development
GO:1901888: regulation of cell junction assembly
GO:0001666: response to hypoxia
GO:0048729: tissue morphogenesis
GO:0030335: positive regulation of cell migration
GO:0060322: head development
GO:0061061: muscle structure development
hsa04926: Relaxin signaling pathway
GO:0099537: trans-synaptic signaling
-log10(P)
SS II vs SS I upregulated
R-HSA-1474244: Extracellular matrix organization
R-HSA-1630316: Glycosaminoglycan metabolism
hsa04080: Neuroactive ligand-receptor interaction
M5884: NABA CORE MATRISOME
hsa04512: ECM-receptor interaction
GO:0044057: regulation of system process
M5885: NABA MATRISOME ASSOCIATED
GO:0044706: multi-multicellular organism process
GO:1902260: negative regulation of delayed rectifier potassium channel activity
GO:0032355: response to estradiol
GO:2000116: regulation of cysteine-type endopeptidase activity
GO:0001944: vasculature development
GO:0007215: glutamate receptor signaling pathway
GO:0030029: actin filament-based process
M65: PID FRA PATHWAY
GO:0030155: regulation of cell adhesion
GO:0010942: positive regulation of cell death
GO:0046328: regulation of JNK cascade
GO:0040017: positive regulation of locomotion
GO:0001819: positive regulation of cytokine production
-log10(P)
c
SS III vs SS I upregulated
R-HSA-1280215: Cytokine Signaling in Immune system
GO:0045087: innate immune response
GO:0098609: cell-cell adhesion
GO:0030155: regulation of cell adhesion
R-HSA-6798695: Neutrophil degranulation
GO:0002703: regulation of leukocyte mediated immunity
GO:0001775: cell activation
M5906: HALLMARK ESTROGEN RESPONSE EARLY
GO:0034330: cell junction organization
hsa05150: Staphylococcus aureus infection
WP3937: Microglia pathogen phagocytosis pathway
GO:0009611: response to wounding
hsa04640: Hematopoietic cell lineage
WP3945: TYROBP causal network in microglia
GO:0060627: regulation of vesicle-mediated transport
GO:0009617: response to bacterium
R-HSA-6809371: Formation of the cornified envelope
GO:0006954: inflammatory response
WP5115: Network map of SARS-CoV-2 signaling pathway
GO:0050878: regulation of body fluid levels
-log10(P)
SS III vs SS II upregulated
GO:0098609: cell-cell adhesion
GO:0034330: cell junction organization
GO:0030855: epithelial cell differentiation
GO:0030155: regulation of cell adhesion
GO:0050878: regulation of body fluid levels
GO:0008544: epidermis development
M5906: HALLMARK ESTROGEN RESPONSE EARLY
GO:0048871: multicellular organismal homeostasis
GO:0048729: tissue morphogenesis
R-HSA-6809371: Formation of the cornified envelope
GO:0000902: cell morphogenesis
M5911: HALLMARK INTERFERON ALPHA RESPONSE
GO:0046903: secretion
GO:0009611: response to wounding
GO:0051046: regulation of secretion
R-HSA-1280215: Cytokine Signaling in Immune system
GO:0007610: behavior
R-HSA-109582: Hemostasis
GO:0120035: regulation of plasma membrane bounded cell projection organization
GO:1903034: regulation of response to wounding
-log10(P)

Figure S7: (a-c). Functional enrichments of six comparisons of DEGs.

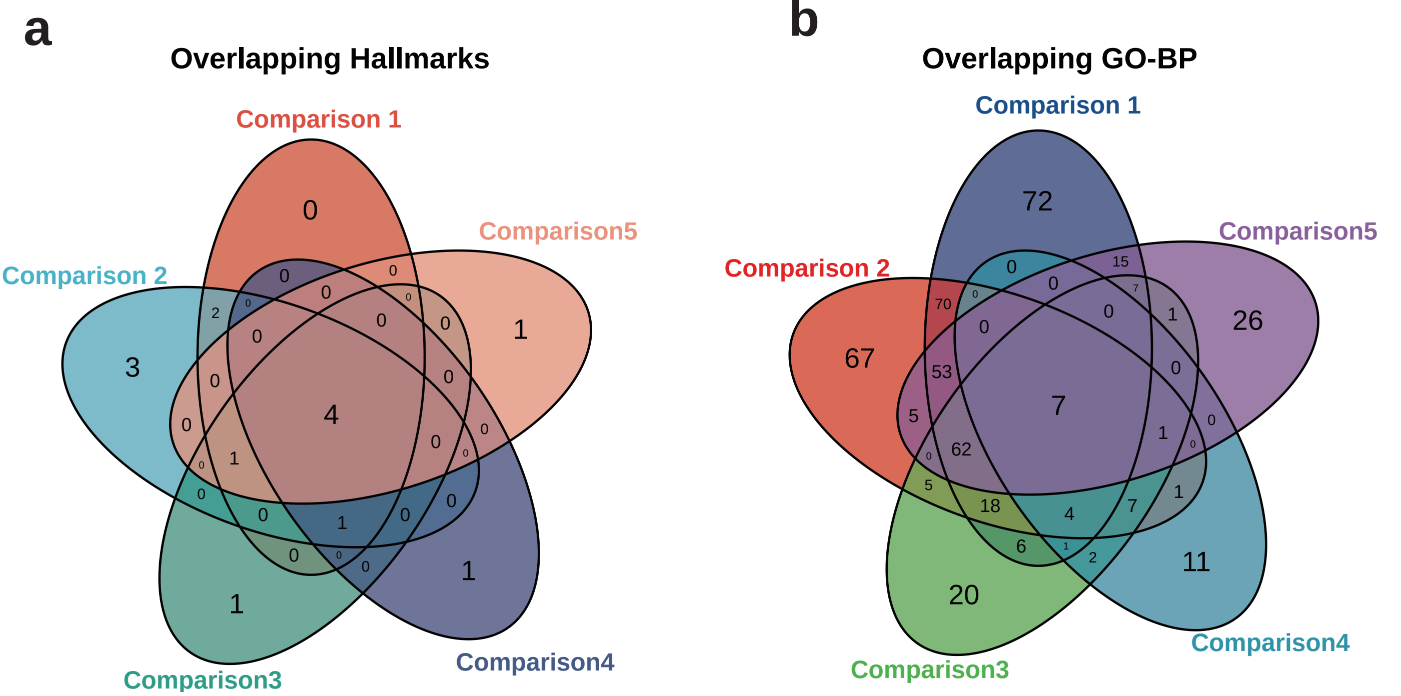

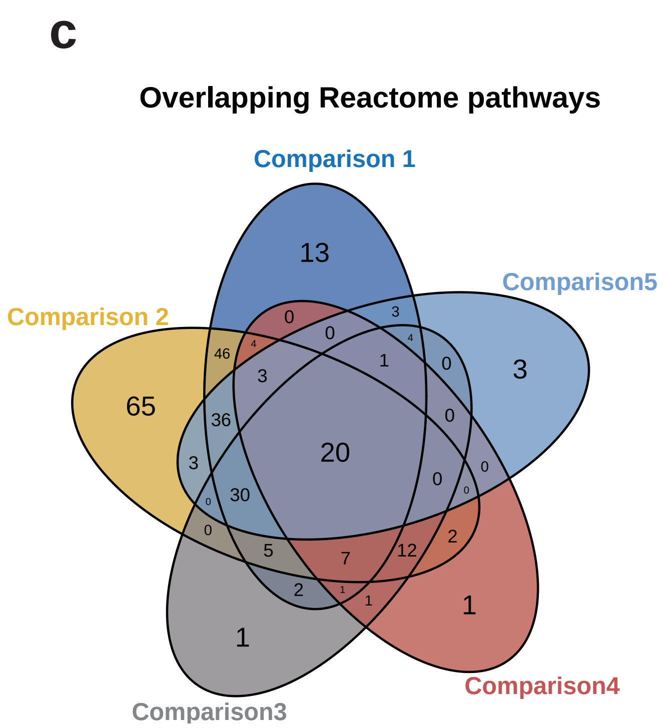

d

Terms	Gene sets
HALLMARK	E2F_TARGETS
HALLMARK	G2M_CHECKPOINT
HALLMARK	MYC_TARGETS_V1
HALLMARK	MYC_TARGETS_V2
GOBP	ATTACHMENT_OF_SPINDLE_MICROTUBULES_TO_KINETOCHORE
GOBP	PROTEIN_LOCALIZATION_TO_CHROMOSOME_CENTROMERIC_REGION
GOBP	POSITIVE_REGULATION_OF_CELL_CYCLE_G2_M_PHASE_TRANSITION
GOBP	DNA_STRAND_ELONGATION_INVOLVED_IN_DNA_REPLICATION
GOBP	MITOCHONDRIAL_TRANSLATION
GOBP	DNA_STRAND_ELONGATION
GOBP	PROTEIN_LOCALIZATION_TO_CHROMOSOME
REACTOME	G2_M_CHECKPOINTS
REACTOME	MITOTIC_SPINDLE_CHECKPOINT
REACTOME	HOMOLOGY_DIRECTED_REPAIR
REACTOME	MITOTIC_G1_PHASE_AND_G1_S_TRANSITION
REACTOME	PROCESSING_OF_DNA_DOUBLE_STRAND_BREAK_ENDS
REACTOME	HOMOLOGOUS_DNA_PAIRING_AND_STRAND_EXCHANGE
REACTOME	RESOLUTION_OF_SISTER_CHROMATID_COHESION
REACTOME	CYCLIN_A_B1_B2_ASSOCIATED_EVENTS_DURING_G2_M_TRANSITION
REACTOME	G2_M_DNA_DAMAGE_CHECKPOINT
REACTOME	S_PHASE
REACTOME	G0_AND_EARLY_G1
REACTOME	NUCLEAR_ENVELOPE_BREAKDOWN
REACTOME	TRANSCRIPTIONAL_REGULATION_BY_SMALL_RNAS
REACTOME	SUMOYLATION_OF_DNA_REPLICATION_PROTEINS
REACTOME	INITIATION_OF_NUCLEAR_ENVELOPE_NE_REFORMATION
REACTOME	MITOTIC_PROPHASE
REACTOME	APC_CDC20_MEDIATED_DEGRADATION_OF_NEK2A
REACTOME	APC_C_CDC20_MEDIATED_DEGRADATION_OF_CYCLIN_B
REACTOME	PHOSPHORYLATION_OF_THE_APC_C
REACTOME	RRNA_PROCESSING

Figure S8: (a-d). Venn diagram depicting the overlap of the overlapping **(a)** Hallmarks, **(b)** GO biological process, and **(c)** Reactome pathways among five comparisons. **(d).** The overlapping gene sets.

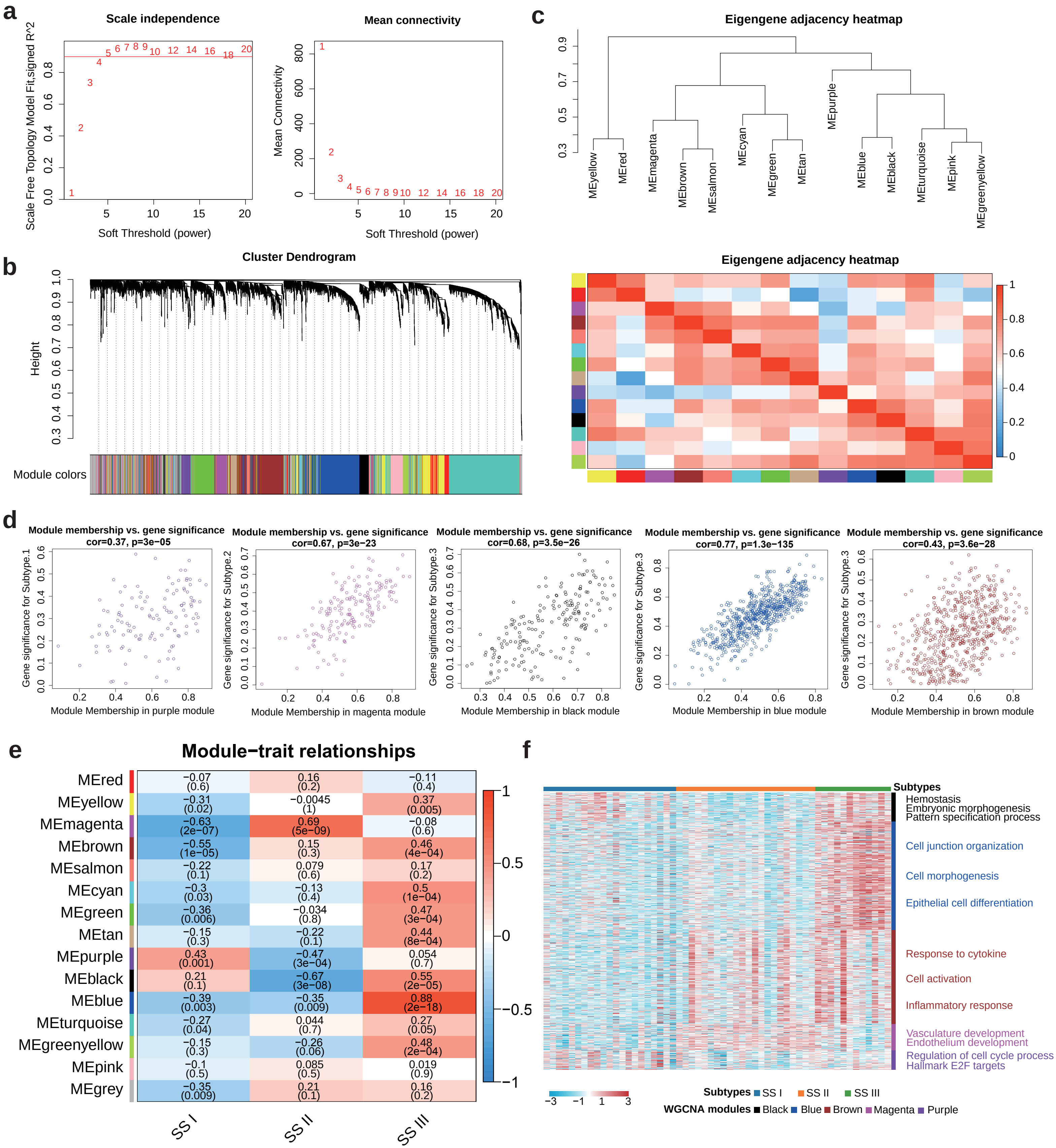
a
Scale independence
Scale Free Topology Model Fit,signed R^2
Soft Threshold (power)
Mean connectivity
Mean Connectivity
Soft Threshold (power)
b
Cluster Dendrogram
Height
Module colors
c
Eigengene adjacency heatmap
MEyellow
MEred
MEmagenta
MEbrown
MEsalmon
MEcyan
MEgreen
MEtan
MEpurple
MEblue
MEblack
MEturquoise
MEpink
MEgreenyellow
Eigengene adjacency heatmap
d
Module membership vs. gene significance
cor=0.37, p=3e−05
Gene significance for Subtype.1
Module Membership in purple module
Module membership vs. gene significance
cor=0.67, p=3e−23
Gene significance for Subtype.2
Module Membership in magenta module
Module membership vs. gene significance
cor=0.68, p=3.5e−26
Gene significance for Subtype.3
Module Membership in black module
Module membership vs. gene significance
cor=0.77, p=1.3e−135
Gene significance for Subtype.3
Module Membership in blue module
Module membership vs. gene significance
cor=0.43, p=3.6e−28
Gene significance for Subtype.3
Module Membership in brown module
e
Module−trait relationships
MEred −0.07 (0.6) 0.16 (0.2) −0.11 (0.4)
MEyellow −0.31 (0.02) −0.0045 (1) 0.37 (0.005)
MEmagenta −0.63 (2e−07) 0.69 (5e−09) −0.08 (0.6)
MEbrown −0.55 (1e−05) 0.15 (0.3) 0.46 (4e−04)
MEsalmon −0.22 (0.1) 0.079 (0.6) 0.17 (0.2)
MEcyan −0.3 (0.03) −0.13 (0.4) 0.5 (1e−04)
MEgreen −0.36 (0.006) −0.034 (0.8) 0.47 (3e−04)
MEtan −0.15 (0.3) −0.22 (0.1) 0.44 (8e−04)
MEpurple 0.43 (0.001) −0.47 (3e−04) 0.054 (0.7)
MEblack 0.21 (0.1) −0.67 (3e−08) 0.55 (2e−05)
MEblue −0.39 (0.003) −0.35 (0.009) 0.88 (2e−18)
MEturquoise −0.27 (0.04) 0.044 (0.7) 0.27 (0.05)
MEgreenyellow −0.15 (0.3) −0.26 (0.06) 0.48 (2e−04)
MEpink −0.1 (0.5) 0.085 (0.5) 0.019 (0.9)
MEgrey −0.35 (0.009) 0.21 (0.1) 0.16 (0.2)
SS I
SS II
SS III
f
Subtypes
Hemostasis
Embryonic morphogenesis
Pattern specification process
Cell junction organization
Cell morphogenesis
Epithelial cell differentiation
Response to cytokine
Cell activation
Inflammatory response
Vasculature development
Endothelium development
Regulation of cell cycle process
Hallmark E2F targets
Subtypes SS I SS II SS III
WGCNA modules Black Blue Brown Magenta Purple

Figure S9: (a). Analysis of the scale-free fit index for various soft-thresholding powers (β). **(b).** Dendrogram of all differentially expressed gene modules clustered based on a dissimilarity measure (1-TOM). **(c).** Adjacency heatmap of the gene modules. **(d).** Scatter plot of module members versus gene significance for SS I in the purple module; SS II in the magenta module; SS III in the black, blue, and brown modules. **(f).** Relationships between WCGNA gene modules and three SS subtypes. Spearman rank correlation. **(g)**. The abundance of five gene modules (black, magenta, blue, purple, and brown) in three SS subtypes and their top functional annotations on the right.

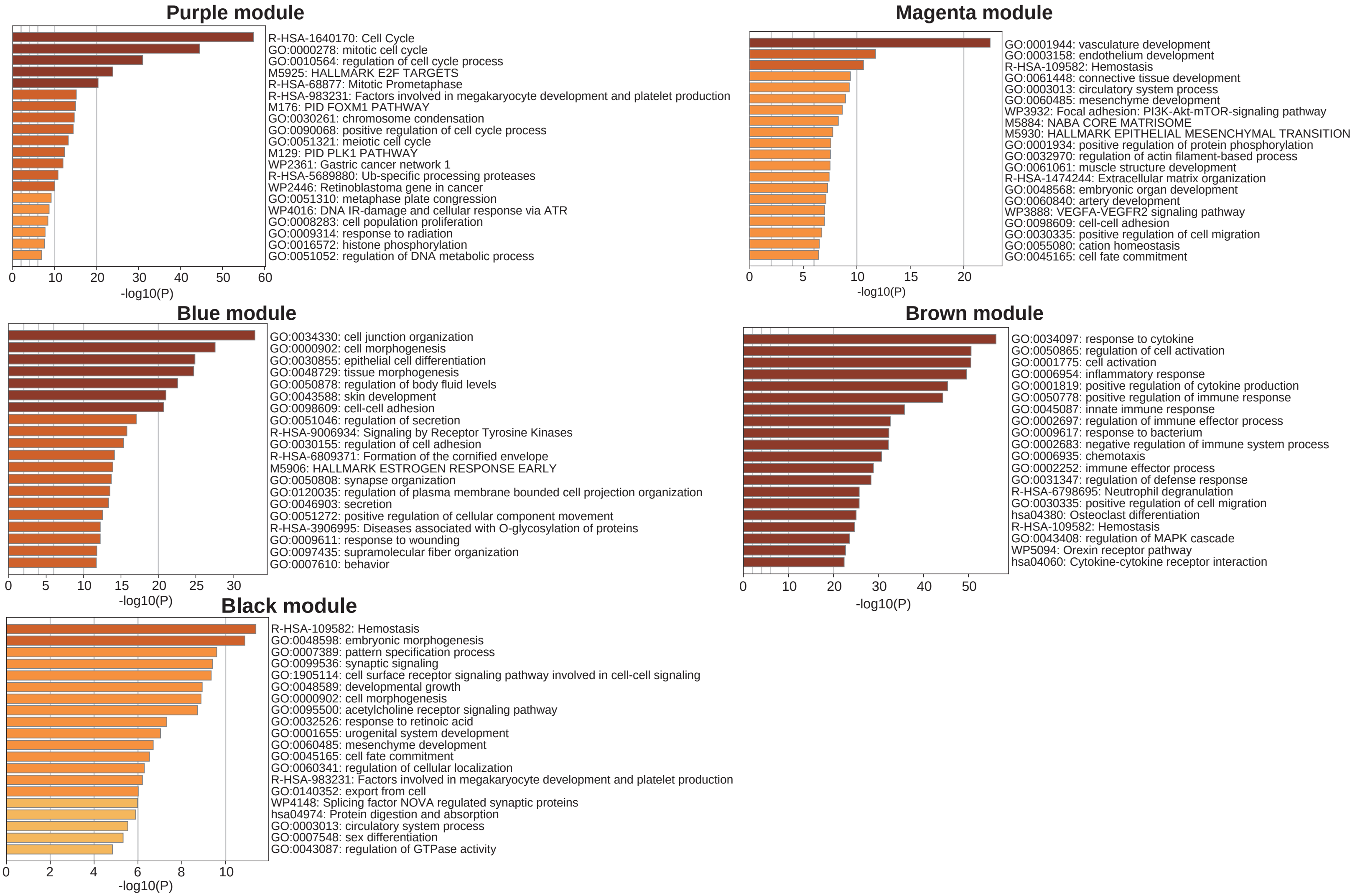
Purple module
R-HSA-1640170: Cell Cycle
GO:0000278: mitotic cell cycle
GO:0010564: regulation of cell cycle process
M5925: HALLMARK E2F TARGETS
R-HSA-68877: Mitotic Prometaphase
R-HSA-983231: Factors involved in megakaryocyte development and platelet production
M176: PID FOXM1 PATHWAY
GO:0030261: chromosome condensation
GO:0090068: positive regulation of cell cycle process
GO:0051321: meiotic cell cycle
M129: PID PLK1 PATHWAY
WP2361: Gastric cancer network 1
R-HSA-5689880: Ub-specific processing proteases
WP2446: Retinoblastoma gene in cancer
GO:0051310: metaphase plate congression
WP4016: DNA IR-damage and cellular response via ATR
GO:0008283: cell population proliferation
GO:0009314: response to radiation
GO:0016572: histone phosphorylation
GO:0051052: regulation of DNA metabolic process
0
10
20
30
40
50
60
-log10(P)
Magenta module
GO:0001944: vasculature development
GO:0003158: endothelium development
R-HSA-109582: Hemostasis
GO:0061448: connective tissue development
GO:0003013: circulatory system process
GO:0060485: mesenchyme development
WP3932: Focal adhesion: PI3K-Akt-mTOR-signaling pathway
M5884: NABA CORE MATRISOME
M5930: HALLMARK EPITHELIAL MESENCHYMAL TRANSITION
GO:0001934: positive regulation of protein phosphorylation
GO:0032970: regulation of actin filament-based process
GO:0061061: muscle structure development
R-HSA-1474244: Extracellular matrix organization
GO:0048568: embryonic organ development
GO:0060840: artery development
WP3888: VEGFA-VEGFR2 signaling pathway
GO:0098609: cell-cell adhesion
GO:0030335: positive regulation of cell migration
GO:0055080: cation homeostasis
GO:0045165: cell fate commitment
0
5
10
15
20
-log10(P)
Blue module
GO:0034330: cell junction organization
GO:0000902: cell morphogenesis
GO:0030855: epithelial cell differentiation
GO:0048729: tissue morphogenesis
GO:0050878: regulation of body fluid levels
GO:0043588: skin development
GO:0098609: cell-cell adhesion
GO:0051046: regulation of secretion
R-HSA-9006934: Signaling by Receptor Tyrosine Kinases
GO:0030155: regulation of cell adhesion
R-HSA-6809371: Formation of the cornified envelope
M5906: HALLMARK ESTROGEN RESPONSE EARLY
GO:0050808: synapse organization
GO:0120035: regulation of plasma membrane bounded cell projection organization
GO:0046903: secretion
GO:0051272: positive regulation of cellular component movement
R-HSA-3906995: Diseases associated with O-glycosylation of proteins
GO:0009611: response to wounding
GO:0097435: supramolecular fiber organization
GO:0007610: behavior
0
5
10
15
20
25
30
-log10(P)
Brown module
GO:0034097: response to cytokine
GO:0050865: regulation of cell activation
GO:0001775: cell activation
GO:0006954: inflammatory response
GO:0001819: positive regulation of cytokine production
GO:0050778: positive regulation of immune response
GO:0045087: innate immune response
GO:0002697: regulation of immune effector process
GO:0009617: response to bacterium
GO:0002683: negative regulation of immune system process
GO:0006935: chemotaxis
GO:0002252: immune effector process
GO:0031347: regulation of defense response
R-HSA-6798695: Neutrophil degranulation
GO:0030335: positive regulation of cell migration
hsa04380: Osteoclast differentiation
R-HSA-109582: Hemostasis
GO:0043408: regulation of MAPK cascade
WP5094: Orexin receptor pathway
hsa04060: Cytokine-cytokine receptor interaction
0
10
20
30
40
50
-log10(P)
Black module
R-HSA-109582: Hemostasis
GO:0048598: embryonic morphogenesis
GO:0007389: pattern specification process
GO:0099536: synaptic signaling
GO:1905114: cell surface receptor signaling pathway involved in cell-cell signaling
GO:0048589: developmental growth
GO:0000902: cell morphogenesis
GO:0095500: acetylcholine receptor signaling pathway
GO:0032526: response to retinoic acid
GO:0001655: urogenital system development
GO:0060485: mesenchyme development
GO:0045165: cell fate commitment
GO:0060341: regulation of cellular localization
R-HSA-983231: Factors involved in megakaryocyte development and platelet production
GO:0140352: export from cell
WP4148: Splicing factor NOVA regulated synaptic proteins
hsa04974: Protein digestion and absorption
GO:0003013: circulatory system process
GO:0007548: sex differentiation
GO:0043087: regulation of GTPase activity
0
2
4
6
8
10
-log10(P)

Figure S10: Functional enrichment of five WGCNA gene modules.

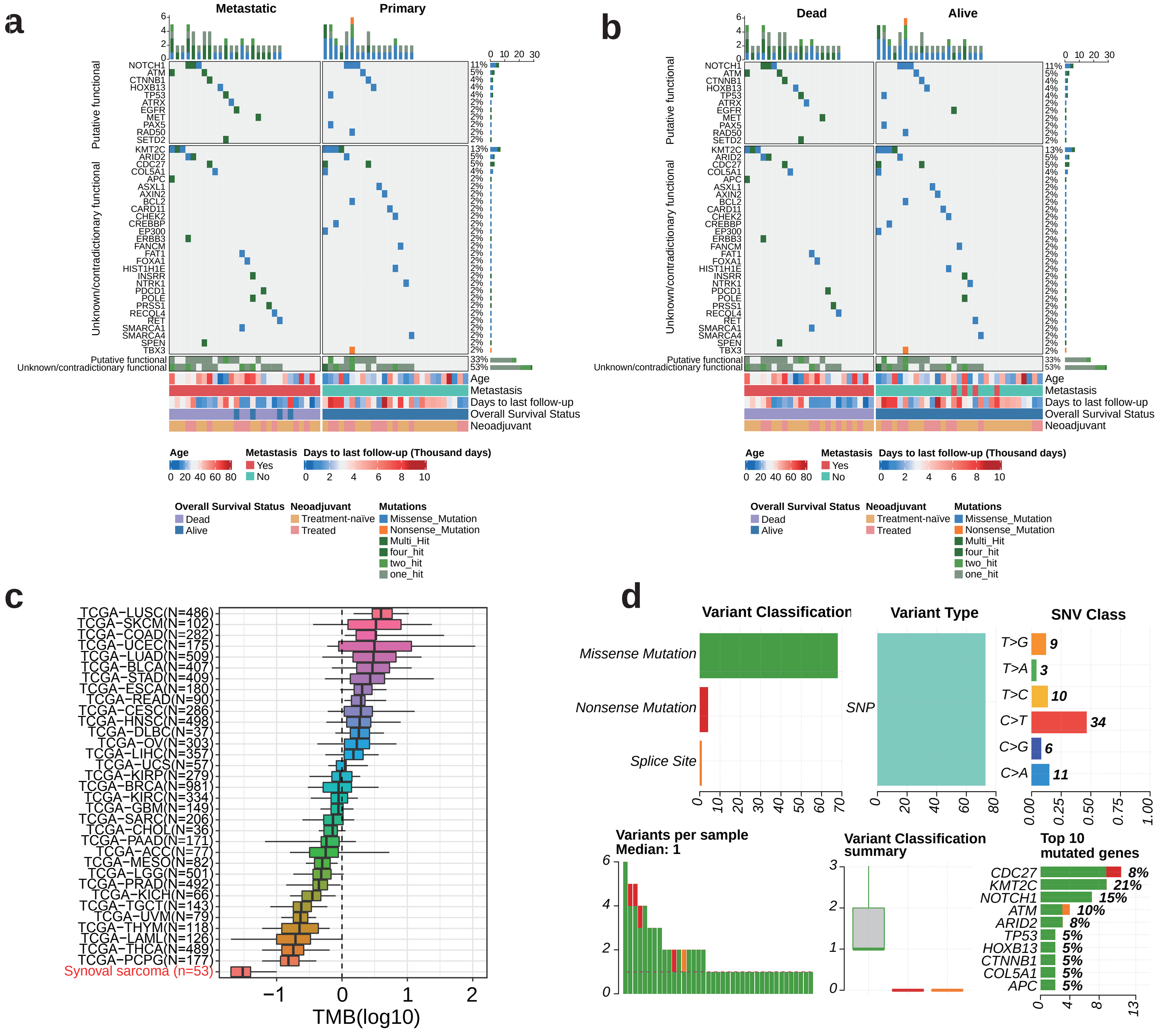

a
Metastatic
Primary
Putative functional
Unknown/contradictionary functional
NOTCH1
ATM
CTNNB1
HOXB13
TP53
ATRX
EGFR
MET
PAX5
RAD50
SETD2
KMT2C
ARID2
CDC27
COL5A1
APC
ASXL1
AXIN2
BCL2
CARD11
CHEK2
CREBBP
EP300
ERBB3
FANCM
FAT1
FOXA1
HIST1H1E
INSRR
NTRK1
PDCD1
POLE
PRSS1
RECQL4
RET
SMARCA1
SMARCA4
SPEN
TBX3
Age
Metastasis
Days to last follow-up
Overall Survival Status
Neoadjuvant
Age
Metastasis
Yes
No
Days to last follow-up (Thousand days)
Overall Survival Status
Dead
Alive
Neoadjuvant
Treatment-naïve
Treated
Mutations
Missense_Mutation
Nonsense_Mutation
Multi_Hit
four_hit
two_hit
one_hit
b
Dead
Alive
c
TCGA−LUSC(N=486)
TCGA−SKCM(N=102)
TCGA−COAD(N=282)
TCGA−UCEC(N=175)
TCGA−LUAD(N=509)
TCGA−BLCA(N=407)
TCGA−STAD(N=409)
TCGA−ESCA(N=180)
TCGA−READ(N=90)
TCGA−CESC(N=286)
TCGA−HNSC(N=498)
TCGA−DLBC(N=37)
TCGA−OV(N=303)
TCGA−LIHC(N=357)
TCGA−UCS(N=57)
TCGA−KIRP(N=279)
TCGA−BRCA(N=981)
TCGA−KIRC(N=334)
TCGA−GBM(N=149)
TCGA−SARC(N=206)
TCGA−CHOL(N=36)
TCGA−PAAD(N=171)
TCGA−ACC(N=77)
TCGA−MESO(N=82)
TCGA−LGG(N=501)
TCGA−PRAD(N=492)
TCGA−KICH(N=66)
TCGA−TGCT(N=143)
TCGA−UVM(N=79)
TCGA−THYM(N=118)
TCGA−LAML(N=126)
TCGA−THCA(N=489)
TCGA−PCPG(N=177)
Synoval sarcoma (n=53)
TMB(log10)
d
Variant Classification
Missense Mutation
Nonsense Mutation
Splice Site
Variant Type
SNP
SNV Class
T>G 9
T>A 3
T>C 10
C>T 34
C>G 6
C>A 11
Variants per sample
Median: 1
Variant Classification summary
Top 10 mutated genes
CDC27 8%
KMT2C 21%
NOTCH1 15%
ATM 10%
ARID2 8%
TP53 5%
HOXB13 5%
CTNNB1 5%
COL5A1 5%
APC 5%

Figure S11: (a-b). Fifty-five synovial sarcoma patients with/without mutation data are ordered by their mutation frequencies and separated by **(a)** metastasis and **(b)** survival status. **(c).** Distributions of tumor mutation burdens of 53 synovial sarcomas and 33 cancer types from TCGA data. Middle line: median; box edges: 25th and 75th percentiles. ACC, adrenocortical carcinoma; BLCA, bladder Urothelial Carcinoma; BRCA, breast invasive carcinoma; CESC, cervical squamous cell carcinoma and endocervical adenocarcinoma; CHOL, cholangiocarcinoma; COAD, colon adenocarcinoma; DLBC, lymphoid neoplasm diffuse large B-cell Lymphoma; ESCA, esophageal carcinoma; GBM, glioblastoma multiforme; HNSC, head and neck squamous cell carcinoma; KICH, kidney chromophobe; KIRC, kidney renal clear cell carcinoma; KIRP, kidney renal papillary cell carcinoma; LAML, acute myeloid leukemia; LGG, brain lower grade glioma; LIHC, liver hepatocellular carcinoma; LUAD, lung adenocarcinoma; LUSC, lung squamous cell carcinoma; MESO, mesothelioma; OV, ovarian serous cystadenocarcinoma; PAAD, pancreatic adenocarcinoma; PCPG, pheochromocytoma and paraganglioma, PRAD, prostate adenocarcinoma; READ, rectum adenocarcinoma; SARC, sarcoma; STAD, stomach adenocarcinoma; SKCM, skin cutaneous melanoma; TGCT, Testicular germ cell tumors; THCA, thyroid carcinoma; THYM, thymoma; UCEC, uterine corpus endometrial carcinoma; UCS, uterine carcinosarcoma; UVM, uveal melanoma. **(d).** Summary of somatic mutation profile in 53 synovial sarcoma patients.

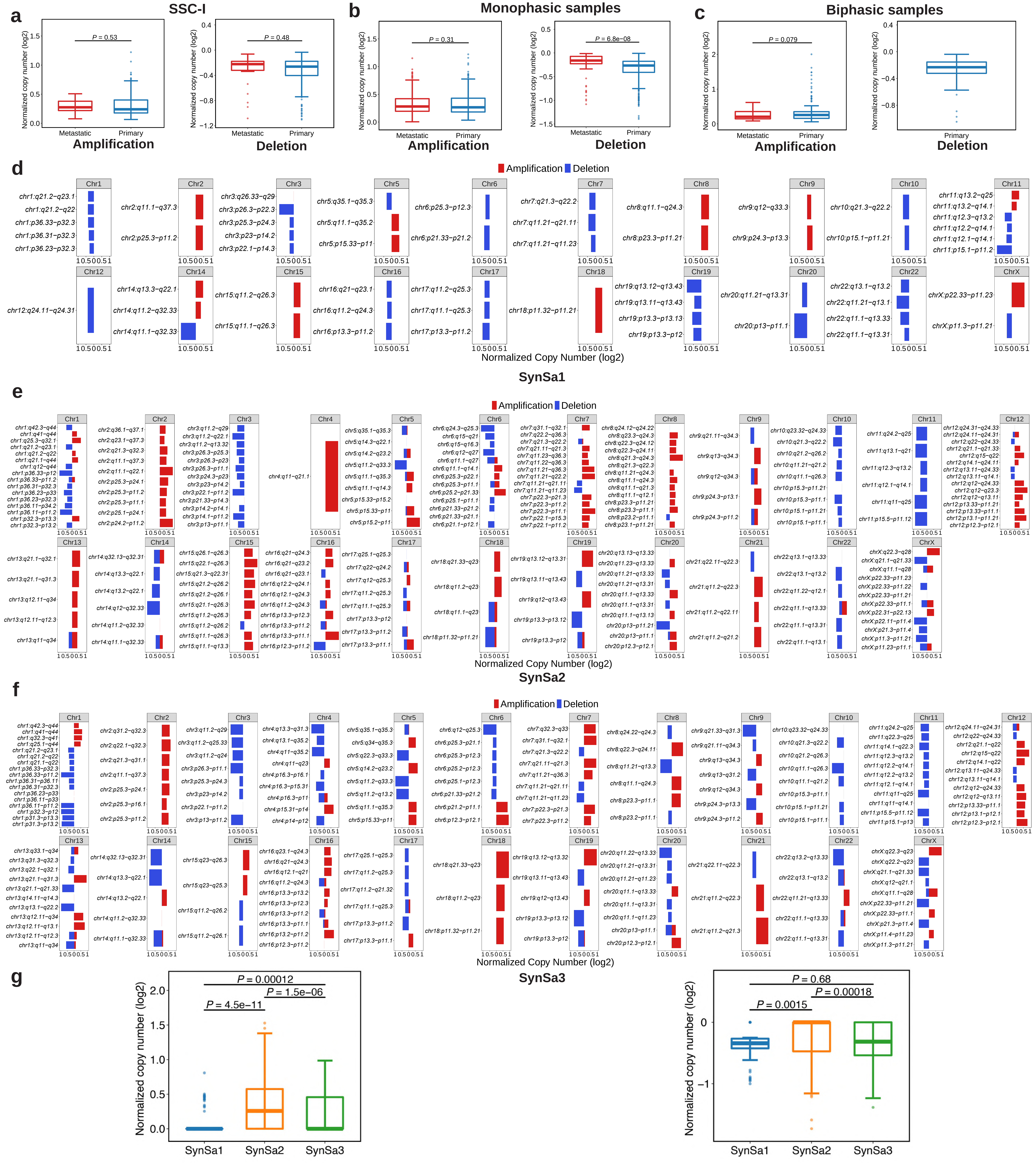
a
SSC-I
P = 0.53
P = 0.48
Normalized copy number (log2)
Metastatic
Primary
Amplification
Deletion
b
Monophasic samples
P = 0.31
P = 6.8e−08
c
Biphasic samples
P = 0.079
d
Amplification Deletion
Normalized Copy Number (log2)
SynSa1
e
SynSa2
f
SynSa3
g
P = 0.00012
P = 1.5e−06
P = 4.5e−11
P = 0.68
P = 0.00018
P = 0.0015
SynSa1
SynSa2
SynSa3

Figure S12: **(a).** Normalized copy number distributions of amplifications and deletions between primary and metastatic in SS I patients. Middle line: median; box edges: 25th and 75th percentiles. Mann-Whitney U test. **(b).** Normalized copy number distributions of amplifications and deletions between primary and metastatic in monophasic samples. Middle line: median; box edges: 25th and 75th percentiles. Mann-Whitney U test. **(c).** Normalized copy number distributions of amplifications and deletions between primary and metastatic in biphasic samples. Middle line: median; box edges: 25th and 75th percentiles. Mann-Whitney U test**. (d-f).** Genome-wide arm-level copy number variations in SynSa1, SynSa2, and SynSa3 groups, respectively. **(g).** Normalized copy number distributions of amplifications and deletions across validation cohort SynSa1, SynSa2 and SynSa3 samples. Middle line: median; box edges: 25th and 75th percentiles. Mann-Whitney U test.

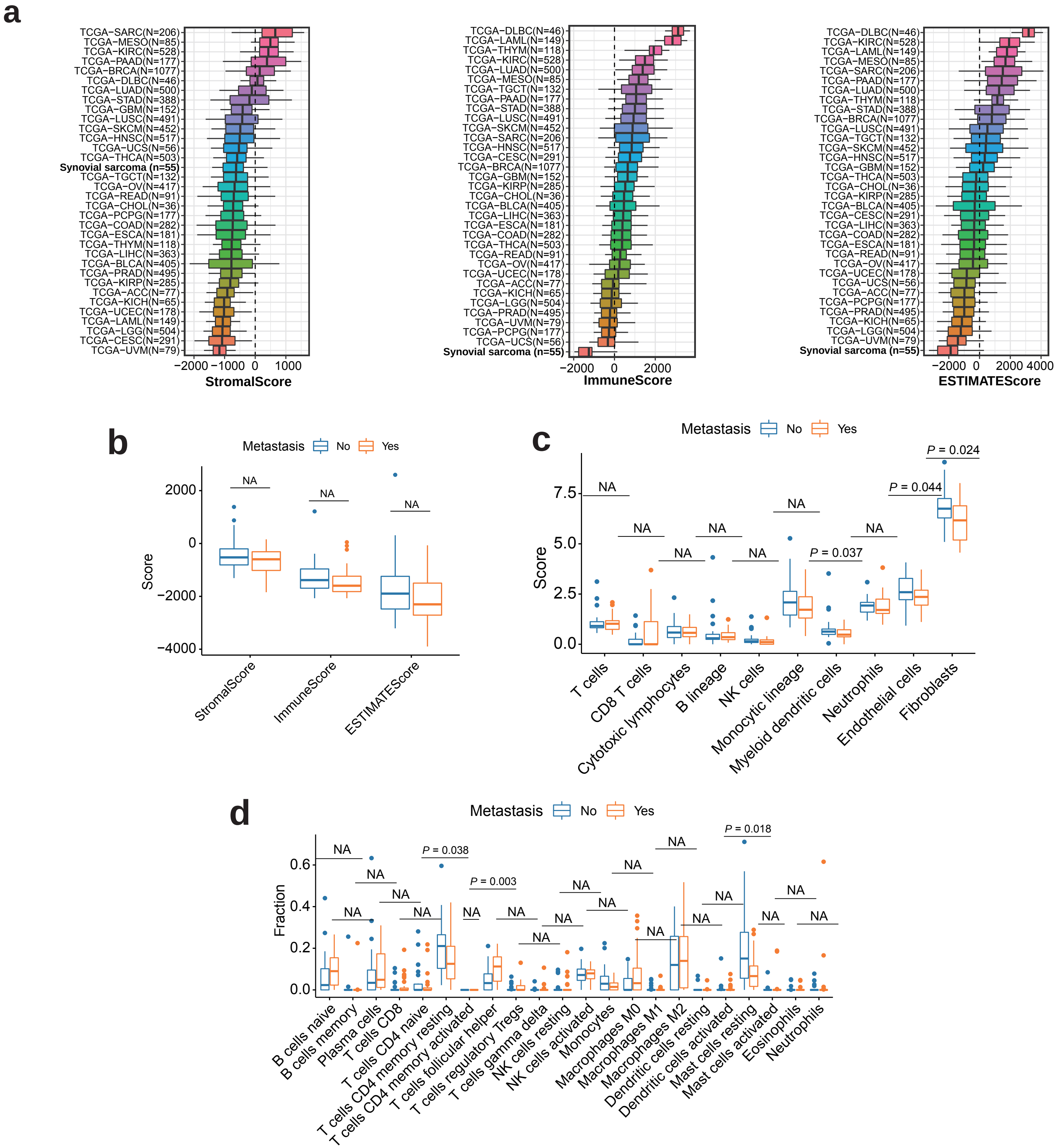
a
TCGA−SARC(N=206)
TCGA−MESO(N=85)
TCGA−KIRC(N=528)
TCGA−PAAD(N=177)
TCGA−BRCA(N=1077)
TCGA−DLBC(N=46)
TCGA−LUAD(N=500)
TCGA−STAD(N=388)
TCGA−GBM(N=152)
TCGA−LUSC(N=491)
TCGA−SKCM(N=452)
TCGA−HNSC(N=517)
TCGA−UCS(N=56)
TCGA−THCA(N=503)
Synovial sarcoma (n=55)
TCGA−TGCT(N=132)
TCGA−OV(N=417)
TCGA−READ(N=91)
TCGA−CHOL(N=36)
TCGA−PCPG(N=177)
TCGA−COAD(N=282)
TCGA−ESCA(N=181)
TCGA−THYM(N=118)
TCGA−LIHC(N=363)
TCGA−BLCA(N=405)
TCGA−PRAD(N=495)
TCGA−KIRP(N=285)
TCGA−ACC(N=77)
TCGA−KICH(N=65)
TCGA−UCEC(N=178)
TCGA−LAML(N=149)
TCGA−LGG(N=504)
TCGA−CESC(N=291)
TCGA−UVM(N=79)
−2000 −1000 0 1000
StromalScore
TCGA−DLBC(N=46)
TCGA−LAML(N=149)
TCGA−THYM(N=118)
TCGA−KIRC(N=528)
TCGA−LUAD(N=500)
TCGA−MESO(N=85)
TCGA−TGCT(N=132)
TCGA−PAAD(N=177)
TCGA−STAD(N=388)
TCGA−LUSC(N=491)
TCGA−SKCM(N=452)
TCGA−SARC(N=206)
TCGA−HNSC(N=517)
TCGA−CESC(N=291)
TCGA−BRCA(N=1077)
TCGA−GBM(N=152)
TCGA−KIRP(N=285)
TCGA−CHOL(N=36)
TCGA−BLCA(N=405)
TCGA−LIHC(N=363)
TCGA−ESCA(N=181)
TCGA−COAD(N=282)
TCGA−THCA(N=503)
TCGA−READ(N=91)
TCGA−OV(N=417)
TCGA−UCEC(N=178)
TCGA−ACC(N=77)
TCGA−KICH(N=65)
TCGA−LGG(N=504)
TCGA−PRAD(N=495)
TCGA−UVM(N=79)
TCGA−PCPG(N=177)
TCGA−UCS(N=56)
Synovial sarcoma (n=55)
−2000 0 2000
ImmuneScore
TCGA−DLBC(N=46)
TCGA−KIRC(N=528)
TCGA−LAML(N=149)
TCGA−MESO(N=85)
TCGA−SARC(N=206)
TCGA−PAAD(N=177)
TCGA−LUAD(N=500)
TCGA−THYM(N=118)
TCGA−STAD(N=388)
TCGA−BRCA(N=1077)
TCGA−LUSC(N=491)
TCGA−TGCT(N=132)
TCGA−SKCM(N=452)
TCGA−HNSC(N=517)
TCGA−GBM(N=152)
TCGA−THCA(N=503)
TCGA−CHOL(N=36)
TCGA−KIRP(N=285)
TCGA−BLCA(N=405)
TCGA−CESC(N=291)
TCGA−LIHC(N=363)
TCGA−COAD(N=282)
TCGA−ESCA(N=181)
TCGA−READ(N=91)
TCGA−OV(N=417)
TCGA−UCEC(N=178)
TCGA−UCS(N=56)
TCGA−ACC(N=77)
TCGA−PCPG(N=177)
TCGA−PRAD(N=495)
TCGA−KICH(N=65)
TCGA−LGG(N=504)
TCGA−UVM(N=79)
Synovial sarcoma (n=55)
−2000 0 2000 4000
ESTIMATEScore
b
Metastasis No Yes
NA
NA
NA
2000
0
−2000
−4000
Score
StromalScore
ImmuneScore
ESTIMATEScore
c
Metastasis No Yes
NA
NA
NA
NA
NA
NA
P = 0.037
NA
P = 0.044
P = 0.024
7.5
5.0
2.5
0.0
Score
T cells
CD8 T cells
Cytotoxic lymphocytes
B lineage
NK cells
Monocytic lineage
Myeloid dendritic cells
Neutrophils
Endothelial cells
Fibroblasts
d
Metastasis No Yes
NA
NA
NA
NA
P = 0.038
NA
NA
P = 0.003
NA
NA
NA
NA
NA
NA
NA
NA
NA
P = 0.018
NA
NA
NA
NA
0.6
0.4
0.2
0.0
Fraction
B cells naive
B cells memory
Plasma cells
T cells CD8
T cells CD4 naive
T cells CD4 memory resting
T cells CD4 memory activated
T cells follicular helper
T cells regulatory Tregs
T cells gamma delta
NK cells resting
NK cells activated
Monocytes
Macrophages M0
Macrophages M1
Macrophages M2
Dendritic cells resting
Dendritic cells activated
Mast cells resting
Mast cells activated
Eosinophils
Neutrophils

Figure S13: (a). Distributions of stromal and immune infiltration abundance by ESTIMATE algorithm of 55 synovial sarcomas and 33 cancer types from TCGA data. **(b-d).** The distributions of immune and stromal cell types between metastatic and primary patients by three deconvolutional approaches, including **(a)** ESTIMATE, **(b)** CIBERSORT, and **(c)** MCPCounter, respectively, Middle line: median; box edges: 25th and 75th percentiles. Mann-Whitney U test.

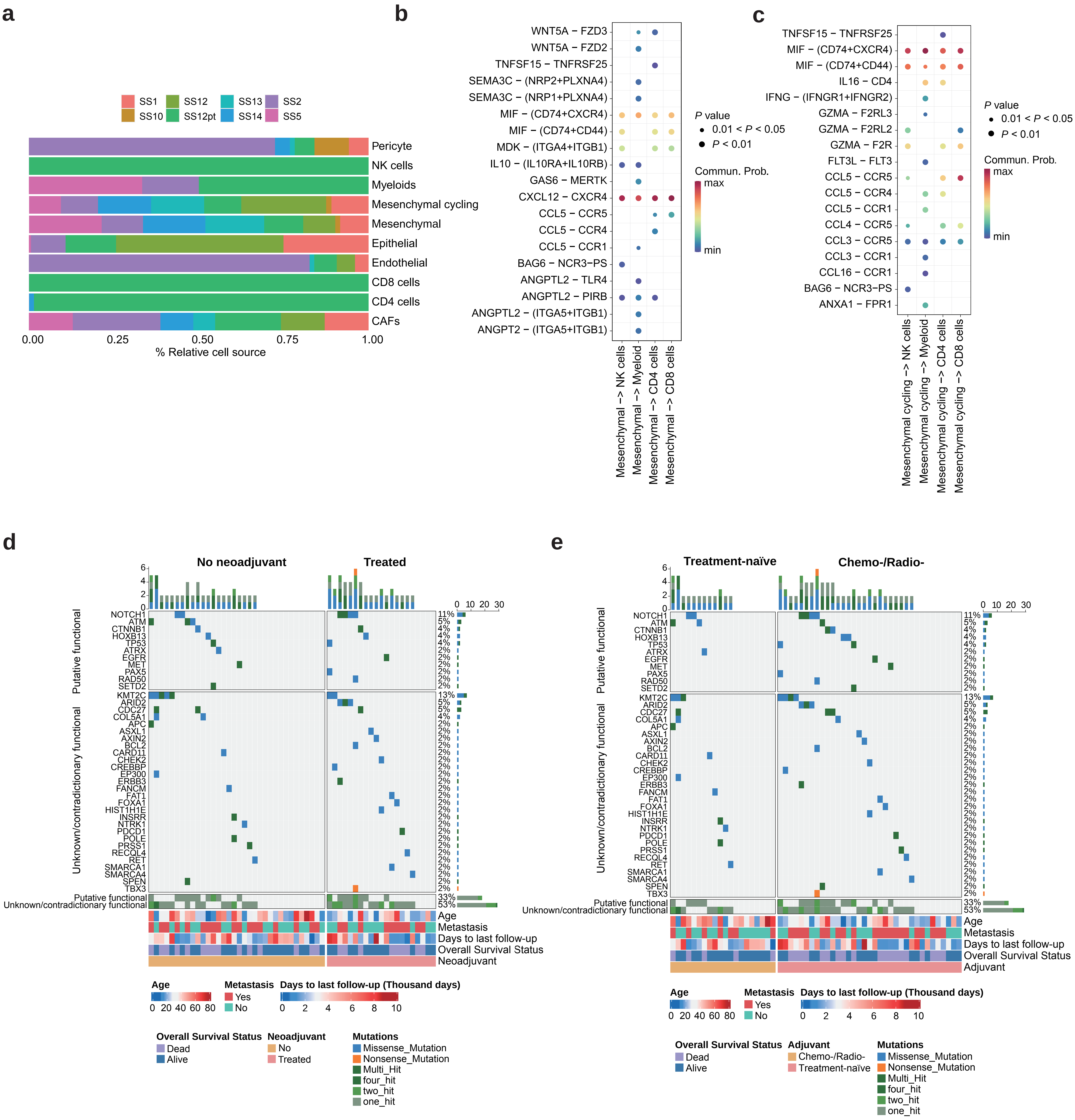
a
SS1
SS12
SS13
SS2
SS10
SS12pt
SS14
SS5
Pericyte
NK cells
Myeloids
Mesenchymal cycling
Mesenchymal
Epithelial
Endothelial
CD8 cells
CD4 cells
CAFs
0.00
0.25
0.50
0.75
1.00
% Relative cell source
b
WNT5A − FZD3
WNT5A − FZD2
TNFSF15 − TNFRSF25
SEMA3C − (NRP2+PLXNA4)
SEMA3C − (NRP1+PLXNA4)
MIF − (CD74+CXCR4)
MIF − (CD74+CD44)
MDK − (ITGA4+ITGB1)
IL10 − (IL10RA+IL10RB)
GAS6 − MERTK
CXCL12 − CXCR4
CCL5 − CCR5
CCL5 − CCR4
CCL5 − CCR1
BAG6 − NCR3−PS
ANGPTL2 − TLR4
ANGPTL2 − PIRB
ANGPTL2 − (ITGA5+ITGB1)
ANGPT2 − (ITGA5+ITGB1)
Mesenchymal −> NK cells
Mesenchymal −> Myeloid
Mesenchymal −> CD4 cells
Mesenchymal −> CD8 cells
P value
0.01 < P < 0.05
P < 0.01
Commun. Prob.
max
min
c
TNFSF15 − TNFRSF25
MIF − (CD74+CXCR4)
MIF − (CD74+CD44)
IL16 − CD4
IFNG − (IFNGR1+IFNGR2)
GZMA − F2RL3
GZMA − F2RL2
GZMA − F2R
FLT3L − FLT3
CCL5 − CCR5
CCL5 − CCR4
CCL5 − CCR1
CCL4 − CCR5
CCL3 − CCR5
CCL3 − CCR1
CCL16 − CCR1
BAG6 − NCR3−PS
ANXA1 − FPR1
Mesenchymal cycling −> NK cells
Mesenchymal cycling −> Myeloid
Mesenchymal cycling −> CD4 cells
Mesenchymal cycling −> CD8 cells
P value
0.01 < P < 0.05
P < 0.01
Commun. Prob.
max
min
d
No neoadjuvant
Treated
Putative functional
Unknown/contradictionary functional
NOTCH1
ATM
CTNNB1
HOXB13
TP53
ATRX
EGFR
MET
PAX5
RAD50
SETD2
KMT2C
ARID2
CDC27
COL5A1
APC
ASXL1
AXIN2
BCL2
CARD11
CHEK2
CREBBP
EP300
ERBB3
FANCM
FAT1
FOXA1
HIST1H1E
INSRR
NTRK1
PDCD1
POLE
PRSS1
RECQL4
RET
SMARCA1
SMARCA4
SPEN
TBX3
11%
5%
4%
4%
4%
2%
2%
2%
2%
2%
2%
13%
5%
5%
4%
2%
33%
53%
0 10 20 30
Age
Metastasis
Days to last follow-up
Overall Survival Status
Neoadjuvant
Age
0 20 40 60 80
Metastasis
Yes
No
Days to last follow-up (Thousand days)
0 2 4 6 8 10
Overall Survival Status
Dead
Alive
Neoadjuvant
No
Treated
Mutations
Missense_Mutation
Nonsense_Mutation
Multi_Hit
four_hit
two_hit
one_hit
e
Treatment-naïve
Chemo-/Radio-
Putative functional
Unknown/contradictionary functional
Age
Metastasis
Days to last follow-up
Overall Survival Status
Adjuvant
Age
0 20 40 60 80
Metastasis
Yes
No
Days to last follow-up (Thousand days)
0 2 4 6 8 10
Overall Survival Status
Dead
Alive
Adjuvant
Chemo-/Radio-
Treatment-naïve
Mutations
Missense_Mutation
Nonsense_Mutation
Multi_Hit
four_hit
two_hit
one_hit

Figure S14: (a). Distributions of the cell proportions in each sample. **(b-c).** Ligand–receptor pairs between **(b)** mesenchymal and **(c)** mesenchymal cycling cells and immune cells (NK cells, myeloid cells, CD4 cells, and CD8 cells. **(d-e).** Fifty-five synovial sarcoma patients with/without mutation data are ordered by their mutation frequencies and separated by **(d)** Neoadjuvant and **(e)** adjuvant treatment status.

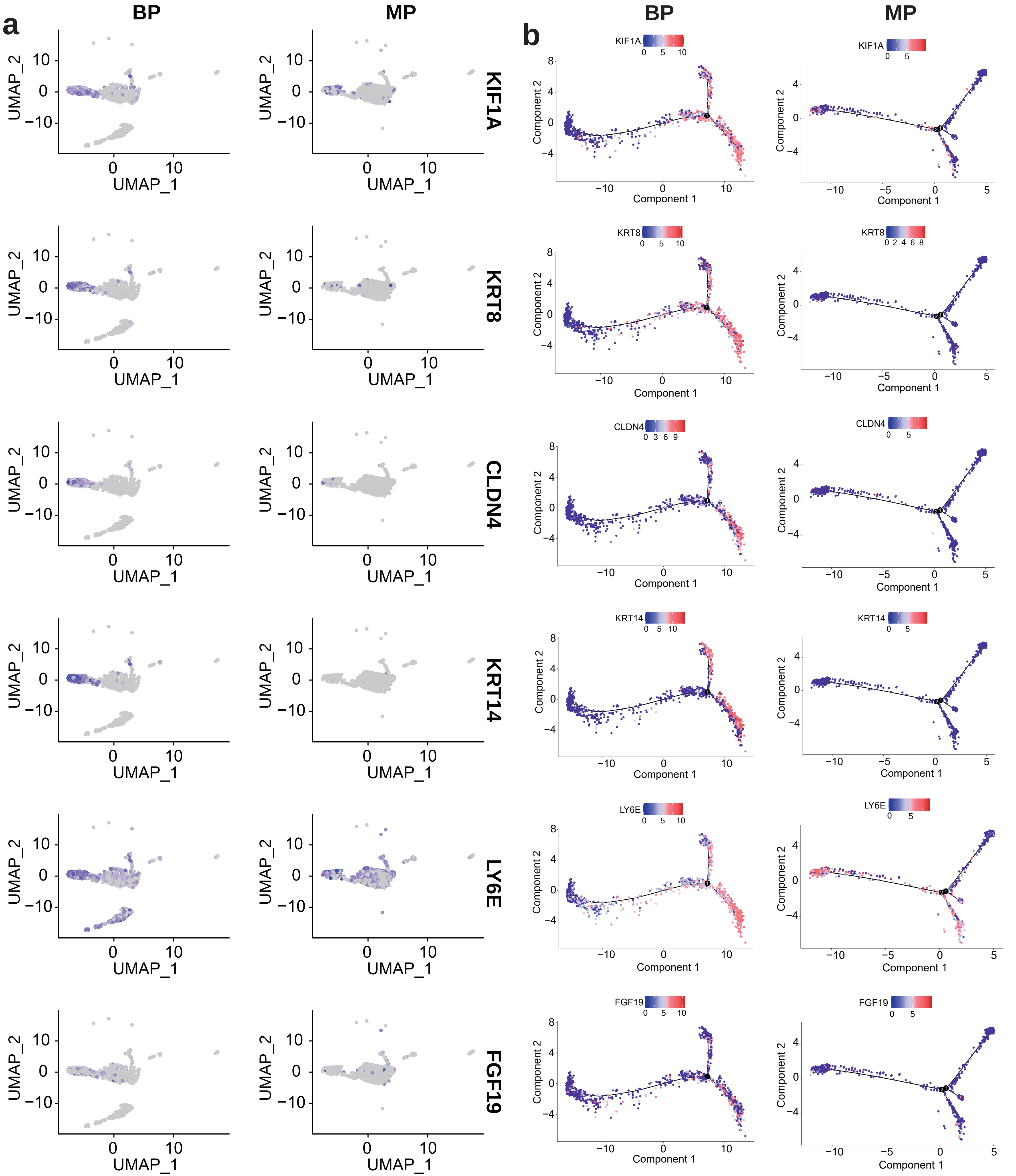
a
b
BP
MP
KIF1A
KRT8
CLDN4
KRT14
LY6E
FGF19
UMAP_1
UMAP_2
Component 1
Component 2

Figure S15: (a). Feature plots for MET key genes (KIF1A, KRT8, CLDN4, KRT14, LY6E, and FGF19) The color legend shows the normalized expression levels of the genes. **(b).** The expression levels of the MET key genes (KIF1A, KRT8, CLDN4, KRT14, LY6E, and FGF19) along with the pseudotime course of the MET process as determined by the Monocle 2 trajectory analysis.

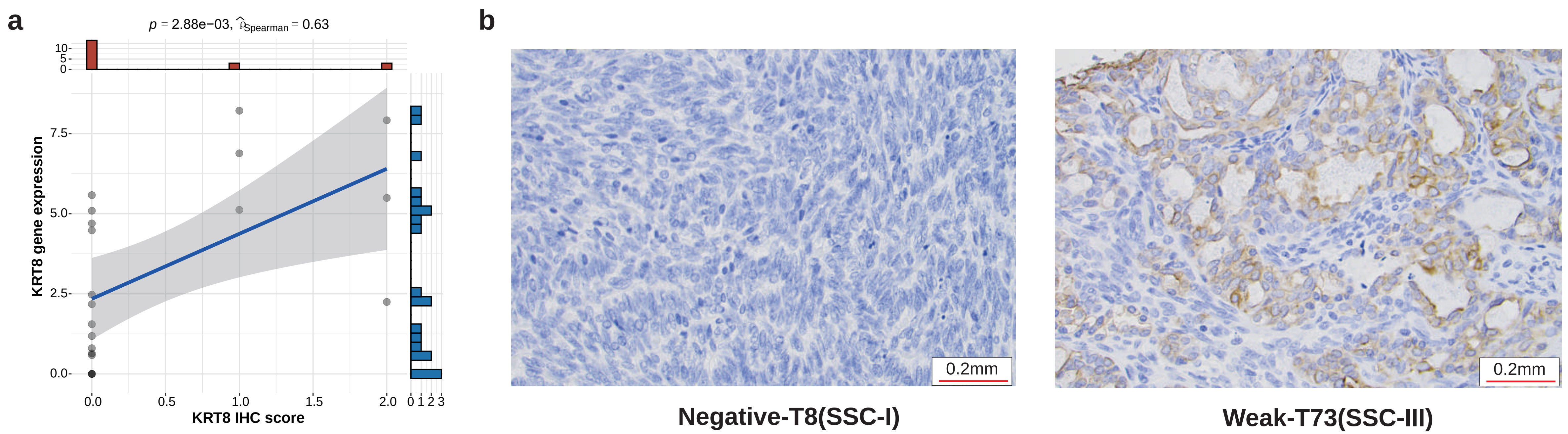

Negative-T8(SSC-I)

Weak-T73(SSC-III)

Figure S16: (a). The correlation between gene expression levels and immunoreactivity of *KRT8*, Spearman rank correlation. **(b).** Immunoreactivity of negative and weak scoring of KRT8 in SS I and SS III, respectively.

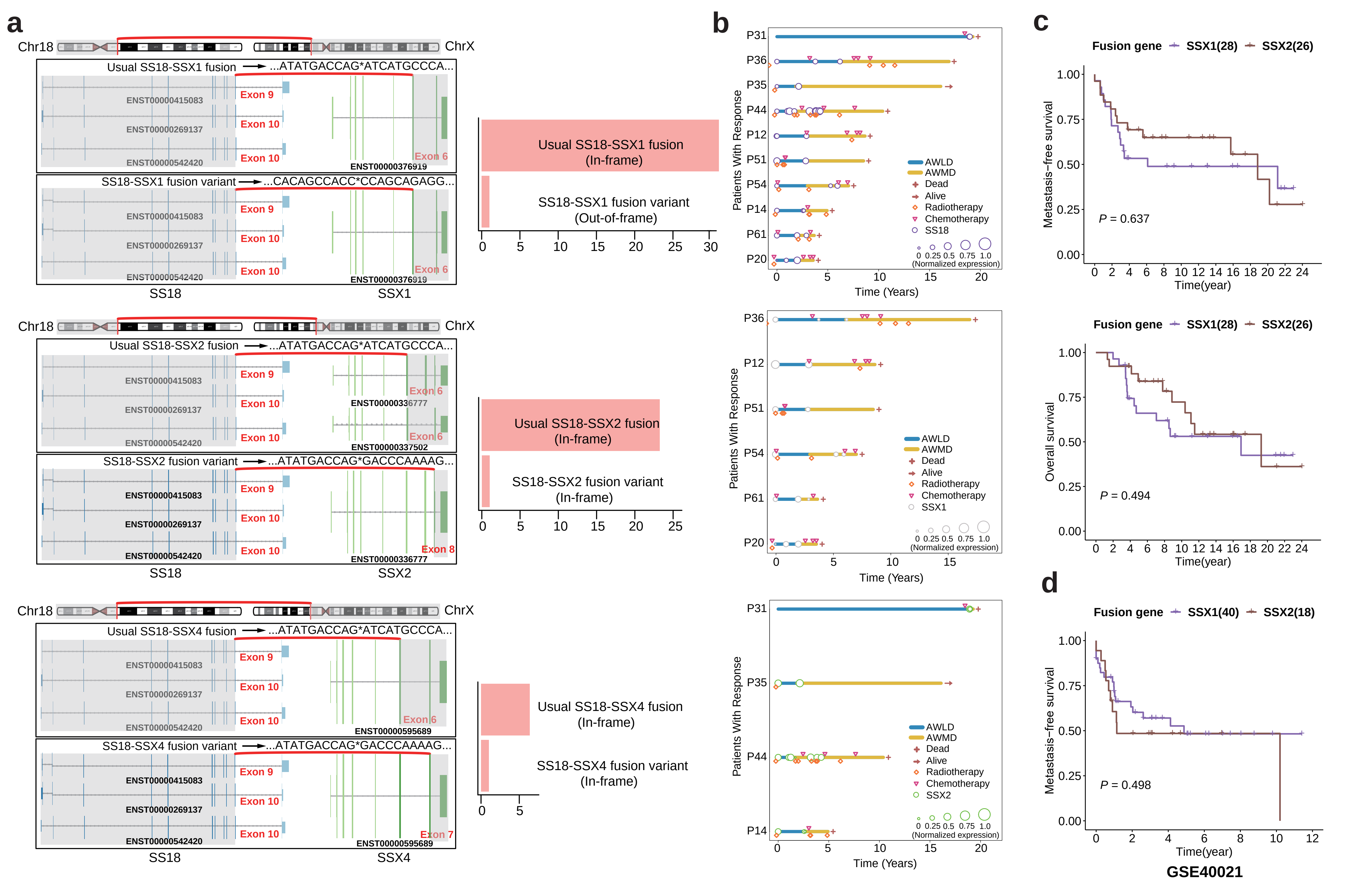
a
Chr18
ChrX
Usual SS18-SSX1 fusion
...ATATGACCAG*ATCATGCCCA...
SS18-SSX1 fusion variant
...CACAGCCACC*CCAGCAGAGG...
ENST00000415083
ENST00000269137
ENST00000542420
ENST00000376919
Exon 9
Exon 10
Exon 6
SS18
SSX1
Usual SS18-SSX1 fusion (In-frame)
SS18-SSX1 fusion variant (Out-of-frame)
Usual SS18-SSX2 fusion
...ATATGACCAG*ATCATGCCCA...
SS18-SSX2 fusion variant
...ATATGACCAG*GACCCAAAAG...
ENST00000336777
ENST00000337502
Exon 8
SSX2
Usual SS18-SSX2 fusion (In-frame)
SS18-SSX2 fusion variant (In-frame)
Usual SS18-SSX4 fusion
...ATATGACCAG*ATCATGCCCA...
SS18-SSX4 fusion variant
...ATATGACCAG*GACCCAAAAG...
ENST00000595689
Exon 7
SSX4
Usual SS18-SSX4 fusion (In-frame)
SS18-SSX4 fusion variant (In-frame)
b
Patients With Response
Time (Years)
AWLD
AWMD
Dead
Alive
Radiotherapy
Chemotherapy
SS18
SSX1
SSX2
(Normalized expression)
c
Fusion gene SSX1(28) SSX2(26)
Metastasis−free survival
Overall survival
Time(year)
P = 0.637
P = 0.494
d
Fusion gene SSX1(40) SSX2(18)
P = 0.498
GSE40021

Figure S17: (a) The landscape of SS18-SSX (1,2,4) variants in the transcripts level and their proportions. **(b).** Assessment of changes in the characteristics of SS18, SSX1, and SSX2 expressions with treatment response in longitudinal analysis. Blue and yellow bars indicate the primary and metastatic status, and the size of circles represents the expression levels, respectively. AWLD, alive with local disease, AWMD, alive with metastatic disease.

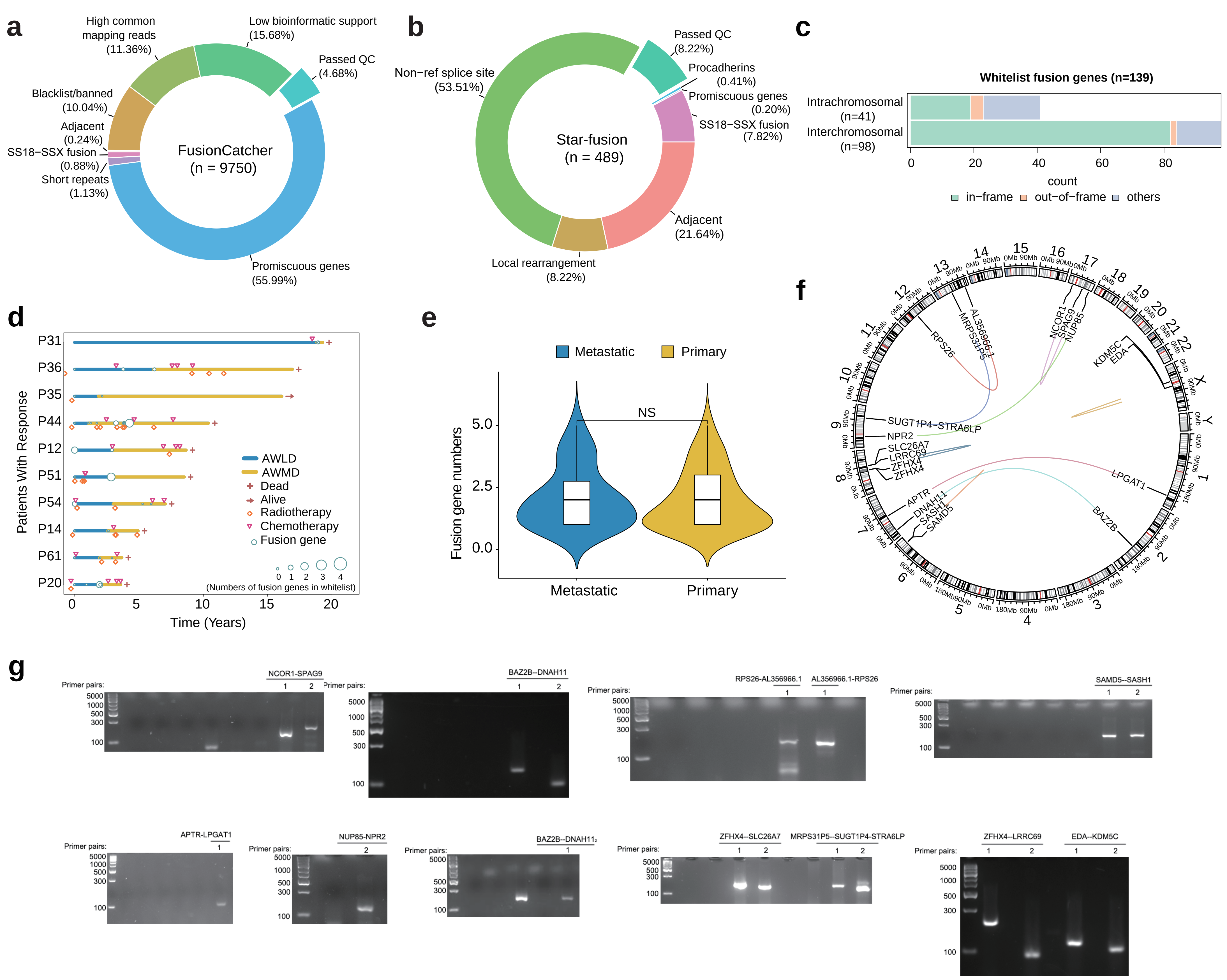

a
FusionCatcher (n = 9750)
High common mapping reads (11.36%)
Low bioinformatic support (15.68%)
Passed QC (4.68%)
Blacklist/banned (10.04%)
Adjacent (0.24%)
SS18−SSX fusion (0.88%)
Short repeats (1.13%)
Promiscuous genes (55.99%)
b
Star-fusion (n = 489)
Non−ref splice site (53.51%)
Passed QC (8.22%)
Procadherins (0.41%)
Promiscuous genes (0.20%)
SS18−SSX fusion (7.82%)
Adjacent (21.64%)
Local rearrangement (8.22%)
c
Whitelist fusion genes (n=139)
Intrachromosomal (n=41)
Interchromosomal (n=98)
count
in−frame
out−of−frame
others
d
Patients With Response
P31
P36
P35
P44
P12
P51
P54
P14
P61
P20
AWLD
AWMD
Dead
Alive
Radiotherapy
Chemotherapy
Fusion gene
(Numbers of fusion genes in whitelist)
Time (Years)
e
Metastatic
Primary
NS
Fusion gene numbers
f
RPS26
MRPS31P5
AL356966.1
NCOR1
SPAG9
NUP85
KDM5C
EDA
SUGT1P4−STRA6LP
NPR2
SLC26A7
LRRC69
ZFHX4
ZFHX4
LPGAT1
APTR
DNAH11
SASH1
SAMD5
BAZ2B
g
Primer pairs:
NCOR1-SPAG9
BAZ2B--DNAH11
RPS26-AL356966.1
AL356966.1-RPS26
SAMD5--SASH1
APTR-LPGAT1
NUP85-NPR2
BAZ2B--DNAH11₂
ZFHX4--SLC26A7
MRPS31P5--SUGT1P4-STRA6LP
ZFHX4--LRRC69
EDA--KDM5C

Figure S18: (a-b). Proportions of fusion gene types generated by **(a)** FusionCatcher and **(b)** Star-fusion. **(c).** Types of the fusion gene in the whitelist. **(d).** Assessment of changes in the characteristics of whitelist fusion gene numbers with treatment response in longitude analysis. Blue and yellow bars indicate the primary and metastatic status, the size of circles represents the expression levels, respectively. **(e).** Distributions of whitelist fusion gene numbers between metastatic and primary samples. Middle line: median; box edges: 25th and 75th percentiles. Mann-Whitney U test. **(f).** Circos plot depicting the validated fusion genes. **(g).** PCR-validated whitelist fusion genes. Labels "1" or "2" denote the distinct clones that were selected from the LB agar plate with the correct size.

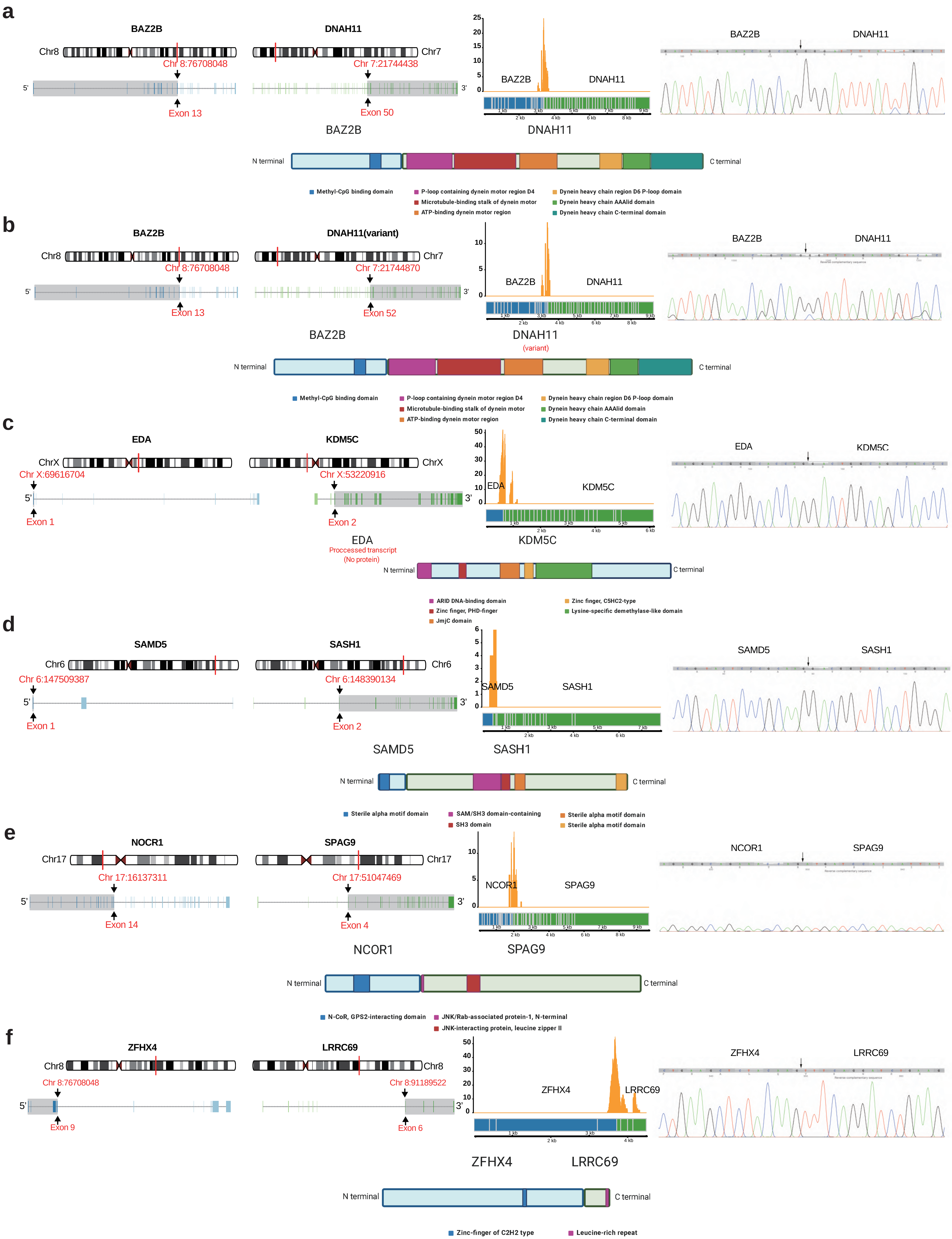
a
BAZ2B
DNAH11
Chr8
Chr7
Chr 8:76708048
Chr 7:21744438
Exon 13
Exon 50
BAZ2B
DNAH11
N terminal
C terminal
Methyl-CpG binding domain
P-loop containing dynein motor region D4
Microtubule-binding stalk of dynein motor
ATP-binding dynein motor region
Dynein heavy chain region D6 P-loop domain
Dynein heavy chain AAAlid domain
Dynein heavy chain C-terminal domain
b
BAZ2B
DNAH11(variant)
Chr8
Chr7
Chr 8:76708048
Chr 7:21744870
Exon 13
Exon 52
BAZ2B
DNAH11
(variant)
N terminal
C terminal
Methyl-CpG binding domain
P-loop containing dynein motor region D4
Microtubule-binding stalk of dynein motor
ATP-binding dynein motor region
Dynein heavy chain region D6 P-loop domain
Dynein heavy chain AAAlid domain
Dynein heavy chain C-terminal domain
c
EDA
KDM5C
ChrX
Chr X:69616704
Chr X:53220916
Exon 1
Exon 2
EDA
Proccessed transcript
(No protein)
KDM5C
N terminal
C terminal
ARID DNA-binding domain
Zinc finger, PHD-finger
JmjC domain
Zinc finger, C5HC2-type
Lysine-specific demethylase-like domain
d
SAMD5
SASH1
Chr6
Chr 6:147509387
Chr 6:148390134
Exon 1
Exon 2
SAMD5
SASH1
N terminal
C terminal
Sterile alpha motif domain
SAM/SH3 domain-containing
SH3 domain
Sterile alpha motif domain
Sterile alpha motif domain
e
NOCR1
SPAG9
Chr17
Chr 17:16137311
Chr 17:51047469
Exon 14
Exon 4
NCOR1
SPAG9
N terminal
C terminal
N-CoR, GPS2-interacting domain
JNK/Rab-associated protein-1, N-terminal
JNK-interacting protein, leucine zipper II
f
ZFHX4
LRRC69
Chr8
Chr 8:76708048
Chr 8:91189522
Exon 9
Exon 6
ZFHX4
LRRC69
N terminal
C terminal
Zinc-finger of C2H2 type
Leucine-rich repeat

Figure S19: (a-f). True positive secondary fusion genes were confirmed by sanger sequencing.
